# Supplementary material for: Genomic clines across the species boundary between a hybrid pine and its progenitor in the eastern Tibetan Plateau
Source: Plant Commun. 2023 Mar 11;4(4):100574. doi: 10.1016/j.xplc.2023.100574 (PMC10363505; doi:10.1016/j.xplc.2023.100574)
Supplement: Document S1. Supplemental Figures 1–4, Supplemental Tables 1, 2, and 6 [file mmc1.pdf]

Plant Communications, Volume *4*

Supplemental information

Genomic clines across the species boundary between a hybrid pine and its progenitor in the eastern Tibetan Plateau

Jing-Fang Guo, Wei Zhao, Bea Andersson, Jian-Feng Mao, and Xiao-Ru Wang

Supplemental Information

Genomic clines across the species boundary between a hybrid pine and its progenitor in the eastern Tibetan Plateau

Jing-Fang Guo, Wei Zhao, Bea Andersson, Jian-Feng Mao, Xiao-Ru Wang

Supplemental information for fastsimcoal analysis

METHOD

As a complementary test to the Stairway Plot approach, we inferred the demographic history of *P. densata* groups (E, C, W, SW) using a coalescent simulation-based method fastsimcoal2 version 2.7 (Excofffier *et al.*, 2021) which takes divergence and migration among populations into consideration. Complex multi-group fastsimcoal models that include migration and population size changes are computationally very time consuming. Previous study (Gao *et al.*, 2012) and our PCA and Stairway results show that group SW and W diverged rather recently, are genetically close and share a similar demography history. Thus to simplify the models, we combined W and SW as one group for this analysis. Because missing data can bias the estimates of SFS, we used a down-sampling procedure to generate the folded SFS using a Python script available on Dryad (Papadopoulou & Knowles, 2015) with minor modification (https://github.com/hui-liu/Bioinformatics-Scripts/blob/master/Scripts/Python/sampleDownMSFS_Hui_final.py). The final folded SFS included low-frequency alleles and invariants.

We tested 11 scenarios, all of which involved the ancestral population splitting into three different groups with gene flow (gene flow was not shown in the Figure 1) but differed in terms of: (i) levels of gene flow between the groups; (ii) the mode of population size change after the split, (iii) whether strong bottleneck occurred or not, and (iv) when the bottleneck occurred (Fig. 1).

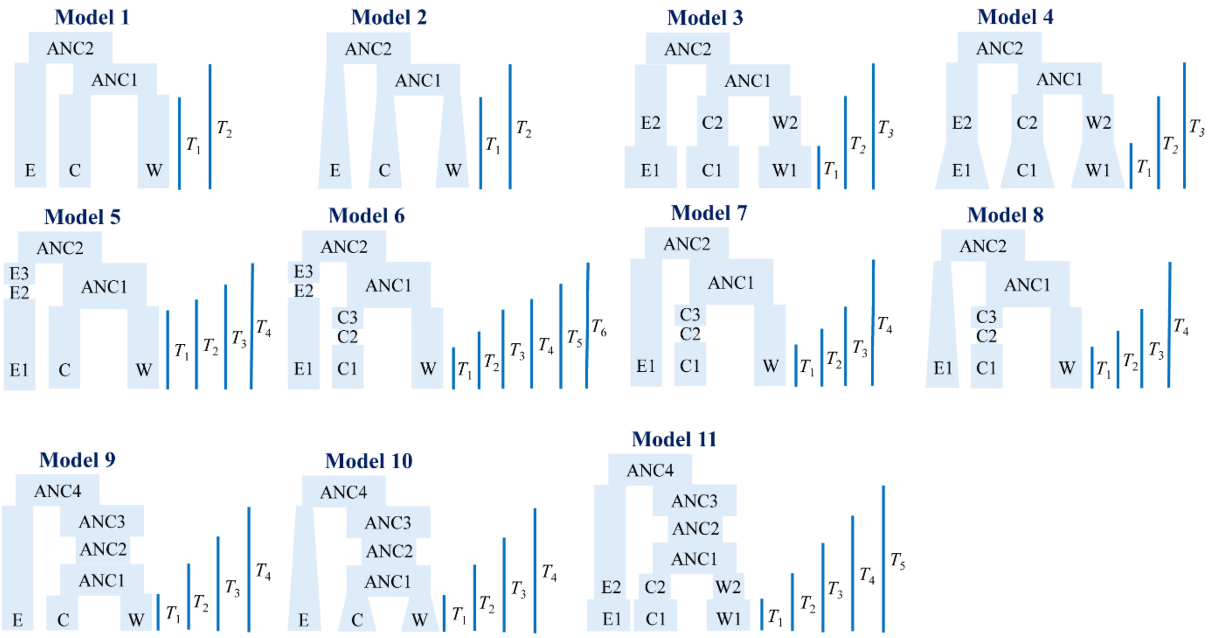

Fig. 1 The 11 tested demographic models.

Each model was run 50 independent times, with 100,000 coalescent simulations as well as 40 expectation-conditional maximization cycles for the calculation of the global maximum-likelihood. The best-fitting model was selected based on the maximum value of likelihood over the 50 independent runs of each modle and their Akaike's weight of evidence (Excoffier *et al.*,

2013). The goodness of fit of the best model was tested by comparing the observed SFS with the expected SFS, which was obtained by 100,000 coalescent simulations under the maximum-likelihood estimates of population parameters. We assumed a mutation rate of 7×10^{-10} mutation per site per year and a generation time of 50 years for the genus *Pinus* (Willyard *et al.*, 2007) to convert model parameters to absolute values.

RESULTS

Of the 11 demographic models, the best-fitting model was model 10. The expected SFS from this model projected the observed SFS well (Fig. 2), suggesting that the demographic parameters recovered are good estimates of the past population history. The model estimated a divergence time of 5.36 million years ago between the E and the ancestor population of group C and W+SW (Fig. 3). The estimated effective population size (N_e) of the common ancestor of the three groups was 1.4×10^4, and N_e of the common ancestor of group C and W+SW was 2.9×10^4. At 0.88–0.58 MYA, this ancestral population of group C and W+SW experienced a strong bottleneck, during which the N_e reduced to half of the original size, then followed by an instantaneous expansion at ~ 0.58 MYA. The group C and W+SW diverged ~ 0.12 MYA accompanied by a bottleneck in each resulting group. The W+SW stared with a very small population size ~1000 individuals. All groups expanded recently.

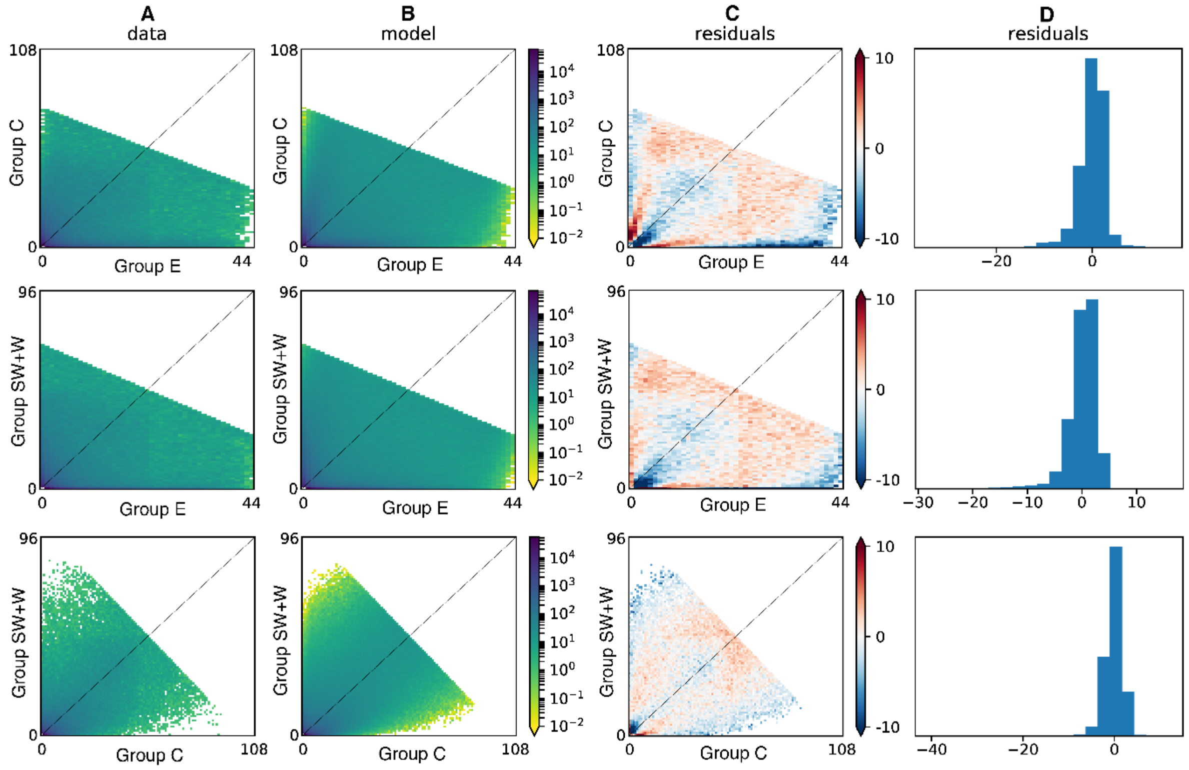

Fig. 2 The goodness of fit of the best-fitting demographic model inferred by fastsimcoal2. For each pair of groups, group E *vs*. C (top), group E *vs*. SW+W (middle) and group C *vs*. SW+W (bottom), the observed joint SFS (data, A) is compared with expected joint SFS (model, B), and the residuals between data and model are plotted in a colormap (C) and a histogram (D). In the colormap, red or blue residuals indicate that the model predicts too many to too few alleles in a given cell, respectively.

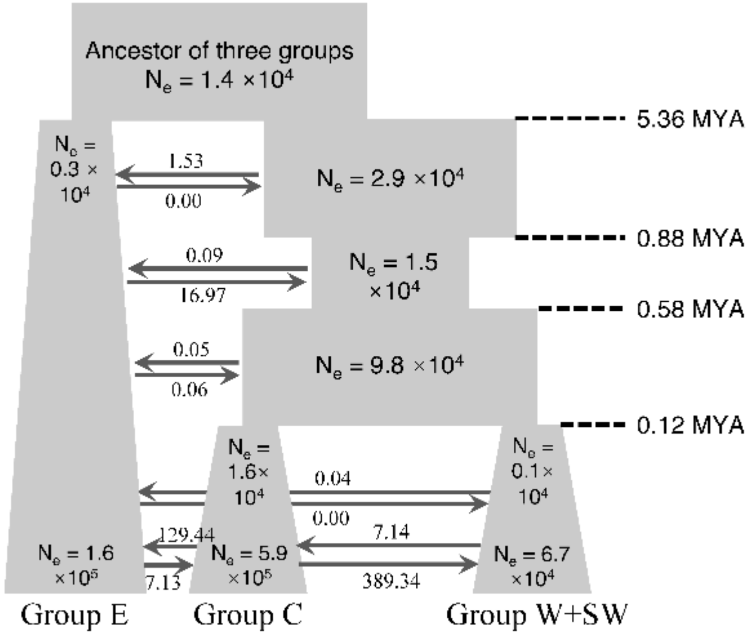

Fig. 3 Demographic model of divergence in *Pinus densata*. Each block represents a current or ancestral population with their estimated effective population size (N_e). Arrows indicate the direction of gene flow with the estimated migration rate labeled above or below the arrow. The timing of population splits and population size changes are indicated in million years ago (MYA).

REFERENCES

Excofffier L, Marchi N, Marques DA, Matthey-Doret R, Gouy A, Sousa VC. 2021. fastsimcoal2: demographic inference under complex evolutionary scenarios. *Bioinformatics* **37**: 4882-4885.

Excoffier L, Dupanloup I, Huerta-Sánchez E, Sousa VC, Foll M. 2013. Robust demographic inference from genomic and SNP data. *PLoS Genetics* **9**: e1003905.

Gao J, Wang B, Mao JF, Ingvarsson P, Zeng QY, Wang XR. 2012. Demography and speciation history of the homoploid hybrid pine *Pinus densata* on the Tibetan Plateau. *Molecular Ecology* **21**: 4811-4827.

Papadopoulou A, Knowles LL. 2015. Genomic tests of the species-pump hypothesis: recent island connectivity cycles drive population divergence but not speciation in Caribbean crickets across the Virgin Islands. *Evolution* **69**: 1501-1517.

Willyard A, Syring J, Gernandt DS, Liston A, Cronn R. 2007. Fossil calibration of molecular divergence infers a moderate mutation rate and recent radiations for *Pinus*. *Molecular Biology and Evolution* **24**: 90-101.

Supplemental Figures

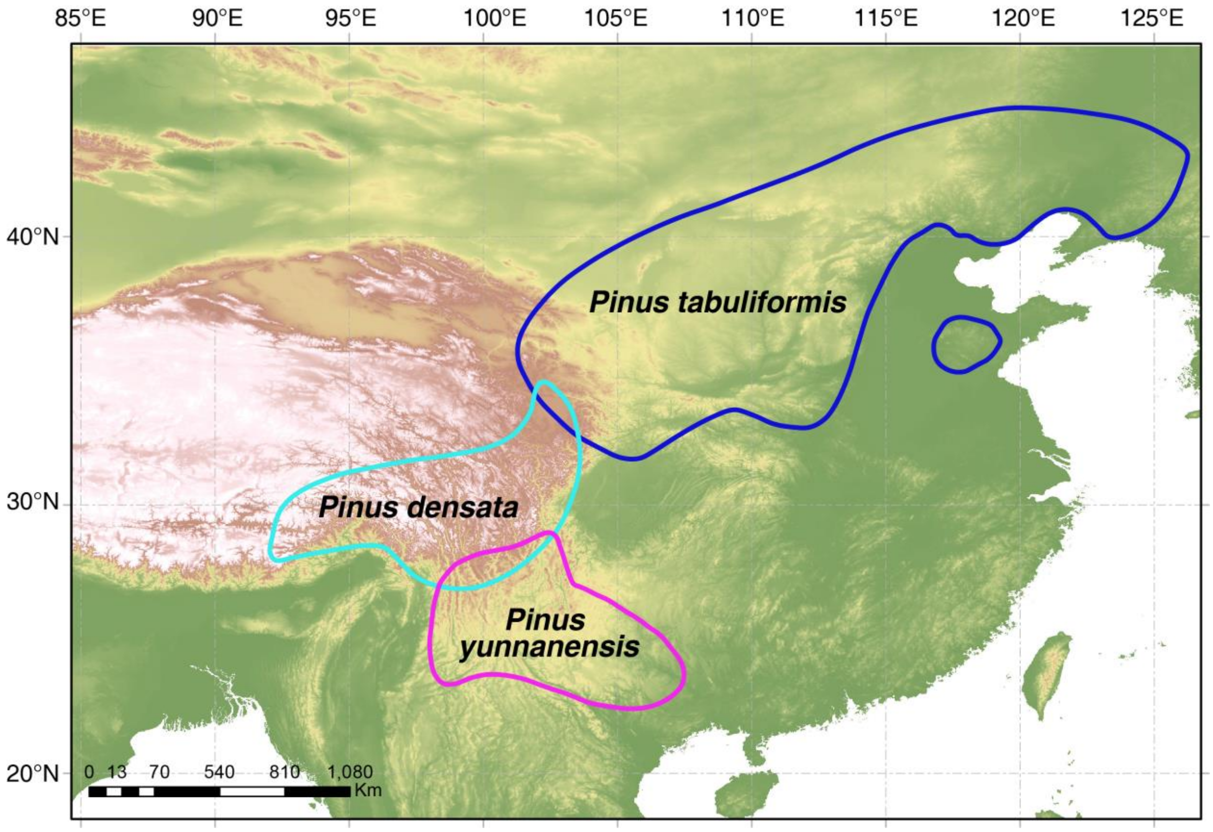

Fig. S1 Distribution map of *Pinus densata*, *P. tabuliformis* and *P. yunnanensis* (adapted from Mao and Wang, 2011, American Naturalist 177:424-439).

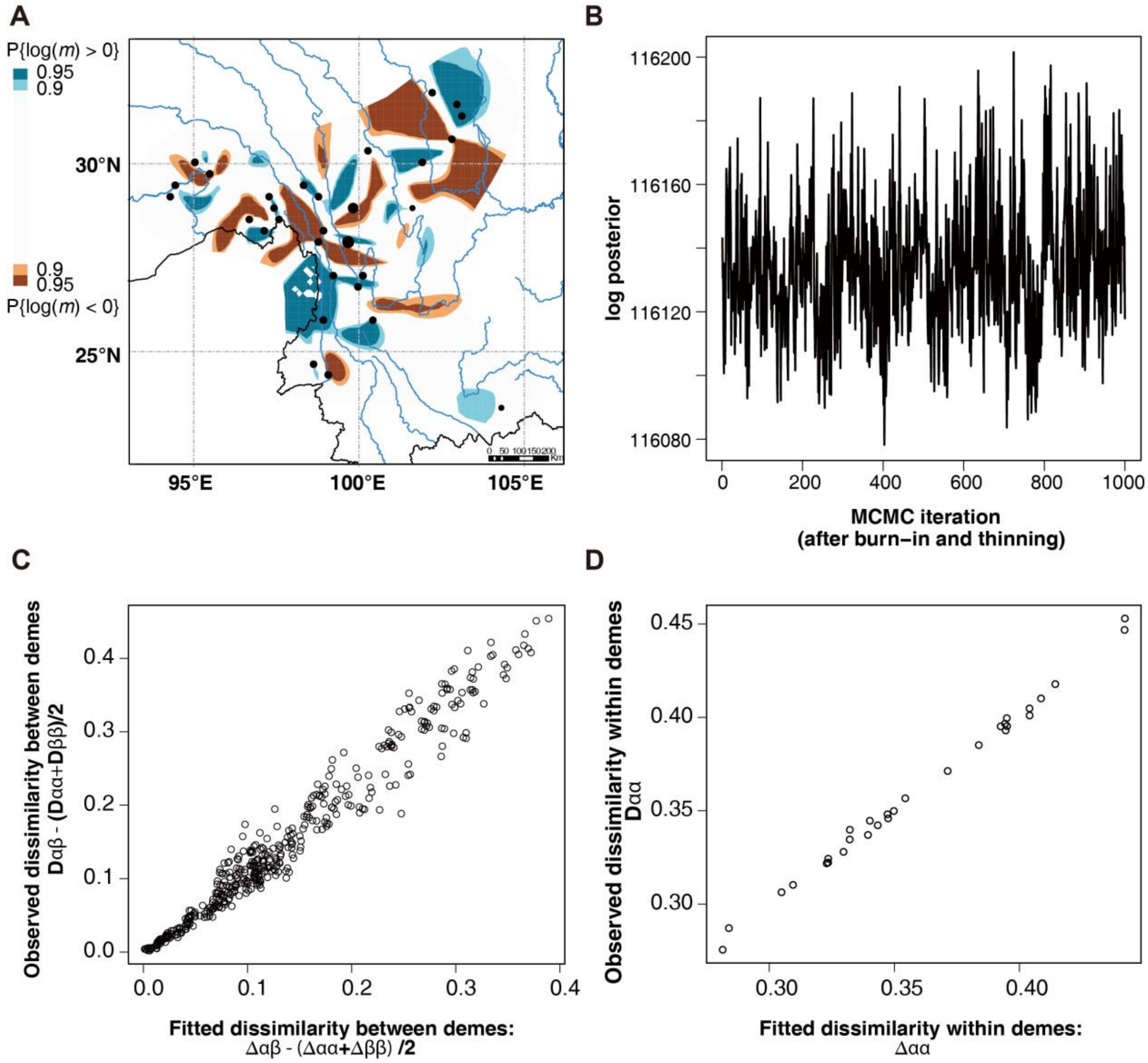

Fig. S2 The reliability of the estimated effective migration surface (EEMS) models. (A) The posterior probabilities of estimated effective migration surface in *Pinus densata* and *P. yunnanensis*. Brown contours represent areas of low relative migration supported by posterior probabilities >0.9. Blue contours represent areas of high relative migration supported by posterior probabilities >0.9. Size of dots reflects numbers of samples in a merged locality. (**B**) Posterior probability trace showing the convergence of EEMS. Observed versus fitted dissimilarities within-demes component (**C**) and between-demes component (**D**). Strong linear relationship between the observed and fitted values indication good fit of EEMS model.

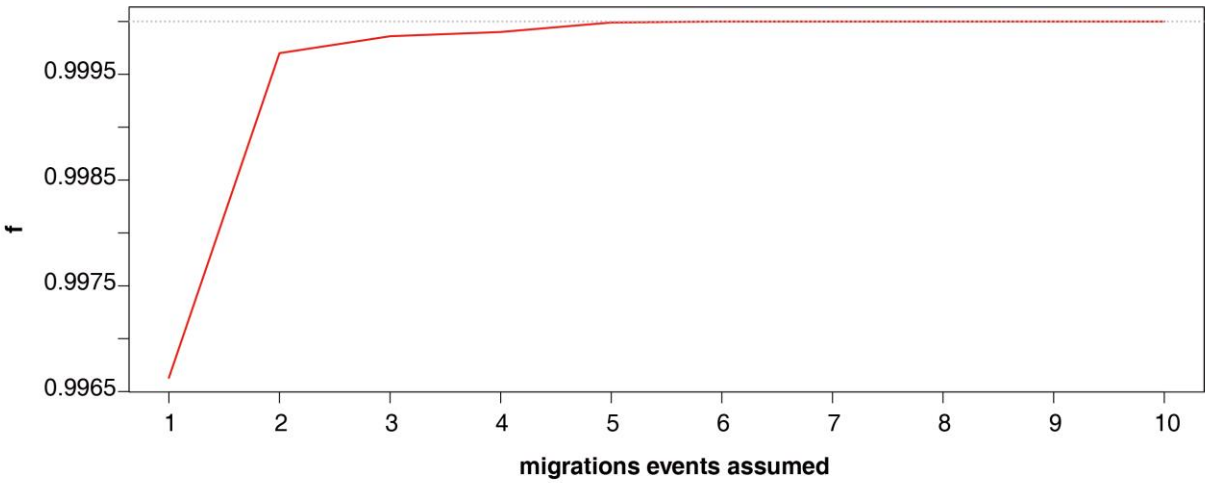

Fig. S3 Measuring models fit in Treemix. Plot showing the *f*-index, which represents the fraction of the variance in the sample covariance matrix explained by the model covariance matrix, as criteria for model fitting for the number of migration events ranging from 1 to 10.

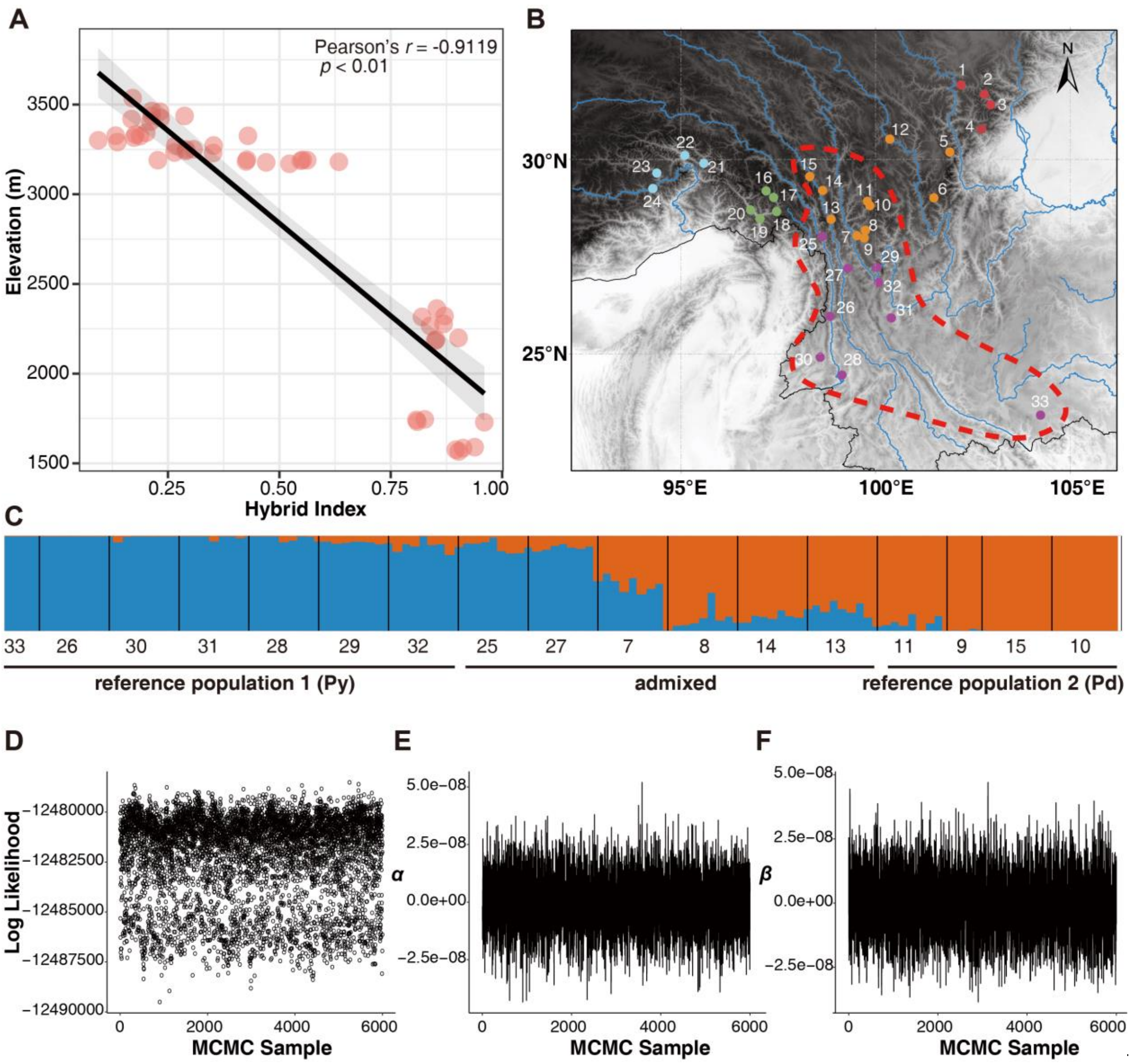

Fig. S4 A zone of species transition selected for Bayesian estimation of genomic clines (BGC) analysis, and the reliability of the BGC models. (A) Correlation of hybrid index of admixed individuals with elevation. **(B)** The distribution of the 17 populations for BGC analysis are circled with red dashes. **(C)** ADMIXTURE analysis of the 17 populations. Inspection of the traces by aggregating three independent runs of BGC. Scatter-plot shows the log likelihood **(D)**, α **(E)**, and β **(F)** of MCMC samples.

Supplemental Tables

Table S1 Differiatation (F_{ST}) between and within the group (E, C, SW and W) of *Pinus densata*.

	Pd-E	Pd-C	Pd-SW	Pd-W
Pd-E	0.0453			
Pd-C	0.2031	0.0269		
Pd-SW	0.2622	0.0832	0.0294	
Pd-W	0.2935	0.1171	0.0966	0.0414

Note: Within-group F_{ST} is in red on the diagonal.

Table S2 Introgression events supported by *D* and f_4 statistics calculated by Dsuite.

P1	P2	P3	*D* statistic	Z-score	*P*-value	*f*4-ratio	BBAA	ABBA	BABA
Pt	E	C	0.0899282	27.3914	0	0.132896	4426.8	4094.13	3418.53
C	SW	Py	0.0494395	10.2406	0	0.270799	3580.85	3672.5	3326.48
Pt	E	Py	0.0845899	24.003	0	0.104286	4509.01	4066.91	3432.54
Pt	E	SW	0.0911228	25.4913	0	0.102746	4467.87	4093.17	3409.5
Pt	E	W	0.0925236	25.231	0	0.093165	4452.32	4086.35	3394.22
W	SW	Py	0.0864658	17.1188	0	0.362799	4352.21	3334.25	2803.54
W	C	Py	0.0271345	4.94543	7.60E-07	0.126251	3774.36	3495.45	3310.76
Py	C	E	0.0200026	4.28036	1.87E-05	0.16918	6850.76	2789.94	2680.51
Py	C	Pt	0.0131173	2.72075	0.00651338	0.0596882	7369.89	2633.77	2565.57
Py	SW	E	0.012839	2.31878	0.0204071	0.104096	7108.39	2659.54	2592.11
Py	W	E	0.0137577	2.10117	0.0356259	0.117647	6715.09	2805.68	2729.53
SW	C	Pt	0.0099593	1.85922	0.0629964	0.043718	7530.55	2540.11	2490.02
W	C	Pt	0.00999736	1.77899	0.0752407	0.0442488	7532.34	2517.35	2467.52
SW	C	E	0.00793074	1.6468	0.0995991	0.07213	6983.96	2668.77	2626.77
W	C	E	0.00634253	1.20105	0.229733	0.0581827	6979.3	2639.56	2606.29
SW	W	C	0.0039803	0.931159	0.351772	0.267623	4180.83	3095.75	3071.2
Py	SW	Pt	0.00362587	0.609817	0.541983	0.0155942	7637.82	2505.63	2487.52
Py	W	Pt	0.00349053	0.52439	0.600007	0.0160729	7241.18	2639.95	2621.59
SW	W	E	0.00186384	0.375315	0.707426	0.0149991	7803.24	2345.15	2336.43
SW	W	Pt	5.86E-05	0.0110131	0.991213	0.000229053	8377.14	2227.3	2227.04

Notes: Only comparisons that are compatible with the inferred phylogenetic relationships and result in positive *D* values are shown.

BBAA: number of sites at which species P1 and P2 shared the derived allele.

ABBA: number of sites at which species P2 and P3 shared the derived allele.

BABA: number of sites at which species P1 and P3 shared the derived allele.

Table S6 Environmental parameters used in this study.

Code	Name	Source	Resultion
bio1	Annual mean air temperature	https://www.climond.org/	30 arc-seconds
bio3	Isothermality	https://www.climond.org/	30 arc-seconds
bio4	Air temperature seasonality	https://www.climond.org/	30 arc-seconds
bio12	Annual precipitation	https://www.climond.org/	30 arc-seconds
bio14	Precipitation of the driest month	https://www.climond.org/	30 arc-seconds
bio15	Precipitation seasonality	https://www.climond.org/	30 arc-seconds
FRS	Ground-frost frequency	http://www.ipcc-data.org/observ/clim/cru_climatologies.html	30 arc-seconds
GDD	Growing degree days	http://nelson.wisc.edu/sage/data-and-models/atlas/maps.php	30 arc-seconds
SC	Soil organic carbon	http://nelson.wisc.edu/sage/data-and-models/atlas/maps.php	30 arc-seconds
WET	Wet-day frequency	http://www.ipcc-data.org/observ/clim/cru_climatologies.html	30 arc-minutes
UVB1	Annual mean UV-B	http://www.ufz.de/gluv/	15 arc-minutes
