# Supplementary material for: Genomic clines across the species boundary between a hybrid pine and its progenitor in the eastern Tibetan Plateau
Source: Plant Commun. 2023 Mar 11;4(4):100574. doi: 10.1016/j.xplc.2023.100574 (PMC10363505; doi:10.1016/j.xplc.2023.100574)
Supplement: Document S3. Article plus supplemental information [file mmc3.pdf]

Plant Communications
Research article

CellPress
Partner Journal

Genomic clines across the species boundary between a hybrid pine and its progenitor in the eastern Tibetan Plateau

Jing-Fang Guo[1,3], Wei Zhao[2,3], Bea Andersson[2], Jian-Feng Mao[1] and Xiao-Ru Wang[2,*]

[1]National Engineering Research Center of Tree Breeding and Ecological Restoration; State Key Laboratory of Tree Genetics and Breeding; Key Laboratory of Genetics and Breeding in Forest Trees and Ornamental Plants, Ministry of Education; College of Biological Sciences and Technology, Beijing Forestry University, Beijing 100083, China

[2]Department of Ecology and Environmental Science, Umeå Plant Science Centre, Umeå University, 90187 Umeå, Sweden

[3]These authors contributed equally to this article.

***Correspondence: Xiao-Ru Wang (xiao-ru.wang@umu.se)**

https://doi.org/10.1016/j.xplc.2023.100574

ABSTRACT

Most species have clearly defined distribution ranges and ecological niches. The genetic and ecological causes of species differentiation and the mechanisms that maintain species boundaries between newly evolved taxa and their progenitors are, however, less clearly defined. This study investigated the genetic structure and clines in *Pinus densata*, a pine of hybrid origin on the southeastern Tibetan Plateau, to gain an understanding of the contemporary dynamics of species barriers. We analyzed genetic diversity in a range-wide collection of *P. densata* and representative populations of its progenitors, *Pinus tabuliformis* and *Pinus yunnanensis*, using exome capture sequencing. We detected four distinct genetic groups within *P. densata* that reflect its migration history and major gene-flow barriers across the landscape. The demographies of these genetic groups in the Pleistocene were associated with regional glaciation histories. Interestingly, population sizes rebounded rapidly during interglacial periods, suggesting persistence and resilience of the species during the Quaternary ice age. In the contact zone between *P. densata* and *P. yunnanensis*, 3.36% of the analyzed loci (57 849) showed exceptional patterns of introgression, suggesting their potential roles in either adaptive introgression or reproductive isolation. These outliers showed strong clines along critical climate gradients and enrichment in a number of biological processes relevant to high-altitude adaptation. This indicates that ecological selection played an important role in generating genomic heterogeneity and a genetic barrier across a zone of species transition. Our study highlights the forces that operate to maintain species boundaries and promote speciation in the Qinghai-Tibetan Plateau and other mountain systems.

Keywords: ecological selection, demographic history, genomic cline, introgression outliers, reproductive isolation, species boundary

Guo J.-F., Zhao W., Andersson B., Mao J.-F., and Wang X.-R. (2023). Genomic clines across the species boundary between a hybrid pine and its progenitor in the eastern Tibetan Plateau. Plant Comm. **4**, 100574.

INTRODUCTION

Mating barriers and adaptive divergence are major drivers of speciation (Mayr, 1942; Nosil et al., 2005). Knowledge about the genetic and ecological causes of species differentiation is thus the key to understanding speciation and the distribution of biodiversity across landscapes and ecosystems. Hybrid zones or secondary contact zones provide opportunities to study contemporary dynamics of selection and evolution of reproductive isolation on a tractable timescale (Barton, 2001; Petit and Excoffier, 2009). Both theoretical and empirical studies suggest that species boundaries can be maintained by pre-mating barriers and post-mating intrinsic genetic incompatibilities between hybridizing species, and/or bounded hybrid superiority in intermediate habitats that maintain restricted hybrid zones and separate the parent populations (Moore, 1977; Barton and Hewitt, 1989; Nosil et al., 2005; Mao and Wang, 2011; Hamilton et al., 2013). Fitness variations along

Published by the Plant Communications Shanghai Editorial Office in association with Cell Press, an imprint of Elsevier Inc., on behalf of CSPB and CEMPS, CAS.

This is an open access article under the CC BY license (http://creativecommons.org/licenses/by/4.0/).

geographical and ecological transitions are expected to generate genomic heterogeneity in introgression, with the direction and rate of introgression influenced by the selective advantage or disadvantage of genetic variants in different genomic and ecological backgrounds (Barton, 2001; Nosil et al., 2009; Gompert and Buerkle, 2012). Genomic heterogeneity can, however, also result from neutral processes such as drift, population structure, and linked selection (Nosil et al., 2009; Cruickshank and Hahn, 2014; Ravinet et al., 2017; Colicchio et al., 2021). One way to disentangle these causes is to compare the frequencies of alleles at each locus with a genome-wide average representing the genomic ancestry of an individual or population, and evaluate genomic clines in rates and patterns of introgression among loci (Gompert and Buerkle, 2012; Fitzpatrick, 2013). Loci of exceptional introgression can be regarded as candidates that might underlie hybrid fitness or assortative mating (Gompert and Buerkle, 2012; Fitzpatrick, 2013). Further association analyses of genomic and environmental clines could provide additional evidence about the role of ecological selection in species delineation and yield important insights into species boundary dynamics and genomic heterogeneity.

Gymnosperms, especially conifers, are mostly outcrossing and show weak genetic incompatibilities even among taxa that diversified more than 10 million years ago (Critchfield, 1975; Zhao et al., 2014). This creates ample opportunities for gene exchange between parapatric or partially overlapping species when pollen dispersal is extensive. Even so, each species has a clearly defined distribution range and ecological niche (Mirov, 1967; Mao and Wang, 2011; Jin et al., 2021), suggesting that intrinsic and extrinsic forces act to define species barriers. However, insights into the forces that operate across species boundaries in the presence of gene flow remain limited.

Pinus densata forms extensive forests in the southeastern Tibetan Plateau (Mao and Wang, 2011). Intermediate forms of its needle and cone traits led to the suggestion that *P. densata* was a hybrid between *Pinus tabuliformis* and *Pinus yunnanensis* (Wu, 1956; Cheng and Fu, 1978). All three species are diploid (2n = 24) and typically wind-pollinated, outcrossing conifers. A series of genetic studies based on mitochondrial (mt), chloroplast (cp), and nuclear DNA provided evidence to support its hybrid origin in the late Miocene (Wang and Szmidt, 1994; Wang et al., 2011; Gao et al., 2012). These studies revealed an ancient hybrid zone between *P. tabuliformis* and *P. yunnanensis* in the northeastern margin of the extant *P. densata* range, where maternal mtDNA haplotypes characteristic of *P. tabuliformis* and *P. yunnanensis* were present, creating high mt haplotype diversity in the region (Song et al., 2002; Wang et al., 2011). From this hybrid zone, *P. densata* migrated westward and colonized its current distribution (Wang et al., 2011; Gao et al., 2012; Zhao et al., 2020). During the westward migration, founder events, allele surfing, and physical environments contributed to distinct population differentiation between geographical regions (Wang et al., 2011; Gao et al., 2012; Zhao et al., 2020). The demographic histories of these populations, which have not yet been studied, would provide additional information about the process of establishment of *P. densata* on the plateau and the impacts of regional geological events on its persistence and genetic diversity.

The three species are currently allopatric, with *P. tabuliformis* distributed in northern China, *P. yunnanensis* in southwest China, and *P. densata* in between them (Supplemental Figure 1). Two contact zones between pairs of these species are located in the southern and northern margins of the *P. densata* distribution, and the contact zone with *P. yunnanensis* is much wider than that with *P. tabuliformis* (Mao and Wang, 2011). The evolutionary history and distribution of this species complex raise an intriguing question: how do newly evolved hybrid species maintain genetic boundaries with their progenitors?

To explore these questions, we performed genome-wide population genetic analyses using exome capture sequencing with 40 000 exome probes in a range-wide collection of *P. densata* populations and representative *P. tabuliformis* and *P. yunnanensis* populations. The choice of genotyping method was dictated by the large (25 Gbp) and highly repetitive genome of pine (Niu et al., 2022), which constrains the feasibility of resequencing-based population studies. Exome capture sequencing enriches the representation of targeted exons while filtering away repetitive regions. We then focused on the broad contact zone between *P. densata* and *P. yunnanensis* to examine genomic patterns of introgression. We aimed to (1) characterize genetic variation over the range of *P. densata* to discern the clinal genetic and gene flow patterns across the landscape, (2) reconstruct the demographic histories of *P. densata* to understand the impact of climate oscillation on population persistence, and (3) quantify patterns of genomic clines across a species transition zone to define the role of selection in maintaining species barriers. Our results show that introgression, geographical barriers to gene flow, persistence during glacial cycles, and ecological selection over heterogeneous landscapes have contributed to the strong population structure in *P. densata* and the buildup of intrinsic barriers with its progenitor. This pattern may be generally applicable to plant speciation and diversification on the Qinghai-Tibetan Plateau (QTP), one of the most prominent biodiversity hotspots on Earth.

RESULTS

Population structure and genetic diversity

We included 24 populations of *P. densata*, two representative populations of *P. tabuliformis*, and nine populations of *P. yunnanensis* in this study (Figure 1A). Exome capture sequencing of 268 sampled trees generated an average of 5.82 million reads per tree, with a mean depth ranging from 17× to 50×. Alignment of the sequence reads to the *Pinus taeda* genome yielded an average of 15.62 Mbp of genomic sequence covered by at least five reads per individual. After stringent filtering, we retained 8 161 108 sites for all 268 individuals. After removal of non-polymorphic sites, 458 321 SNPs were retained; a further minor allele frequency (MAF) <0.05 filtering reduced the final dataset to 79 028 SNPs. Exclusion of *Pinus sylvestris* (outgroup) samples yielded 77 368 SNPs (MAF >0.05) in the 260 samples of *P. tabuliformis*, *P. yunnanensis*, and *P. densata*.

FastSTRUCTURE determined $K = 5$ to be an optimal number of clusters to explain the genetic structure in the *P. densata* species complex (Figure 1B). Similar to the report of Zhao et al. (2020), one cluster was unique to *P. yunnanensis*, and the other four

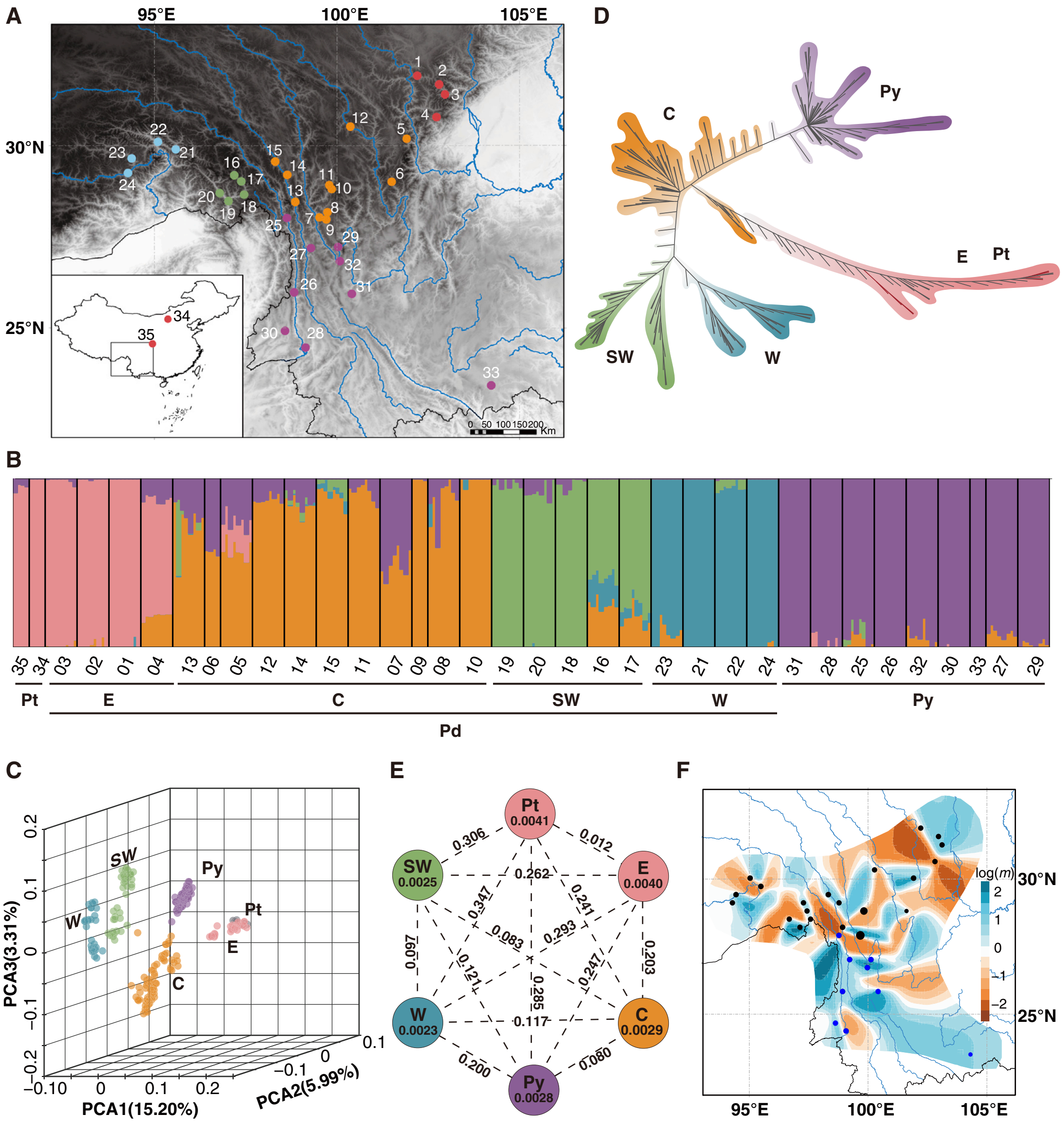

Figure 1. Distribution of sampled populations and their structure, relationships, and diversity

(A) Geographic distribution of 24 sampled populations of *P. densata*, nine representative populations of *P. yunnanensis*, and two populations of *P. tabuliformis*.

(B) Population structure estimated by fastSTRUCTURE for 35 populations of *P. tabuliformis* (Pt), *P. yunnanensis* (Py), and *P. densata* (Pd) at $K = 5$. Each bar represents an individual, and the lengths of colored segments represent the proportions of the genome from different ancestries. Four groups were visible in Pd: east (E), central (C), southwest (SW), and west (W).

(C) Principal-component analysis (PCA) was used to explore the genetic differentiation of Pt, Py, and groups of Pd, with different colors representing different species/groups.

(D) Neighbor-joining (NJ) tree showing relationships among the samples.

(E) Nucleotide diversity (π; values in each circle) and pairwise differentiation ($F_{ST;}$ values on each line) between species/groups.

(F) Estimated effective migration surface (*m*) as inferred by EEMS in Py (blue sampling points) and Pd (black sampling points). Blue colors represent areas of high migration corridors, whereas orange regions represent areas of low migration or dispersal barriers. The map at the bottom left of **(A)** was downloaded from the National Catalogue Service for Geographic Information (www.webmap.cn).

clusters split *P. densata* into distinct geographic zones: west (W), southwest (SW), central (C), and east (E). The eastern range of *P. densata* was dominated by *P. tabuliformis* ancestry (Figure 1B). This grouping was further supported by principal-component analysis (PCA), showing clear division of group E and *P. tabuliformis* from the other four groups/species along PC1, and separations of the other groups/species along PC2 and PC3. The first three PCs jointly explained 24.50% of the total variation (Tracy–Widom test, $p < 0.001$, Figure 1C). A neighbor-joining (NJ) tree also recovered a topology that assigned samples to five major groups with admixed individuals occupying intermediate positions along branches (Figure 1D).

Differentiation among the four groups of *P. densata* (E, C, W, and SW) was significant, with F_{ST} between the groups ranging from 0.083 to 0.294 with an average value of 0.176, which was 5-fold higher than the average F_{ST} (0.036) between populations within each group (Supplemental Table 1). The highest F_{ST} value was observed between the peripheries of W and E. Notably, nucleotide diversity (π) in each group of *P. densata* decreased gradually from east (π = 0.0040) to west (π = 0.0023) (Table 1, Figure 1E). Tajima's *D* for each group was negative, which indicated population expansion in the recent past (Table 1).

The high F_{ST} values among geographical regions motivated us to generate an overview of gene flow across the landscape. The estimated effective migration surface (EEMS) highlighted scattered barriers to gene flow (Figure 1F) that mostly coincided with population subdivision. One strong barrier in the eastern part of the *P. densata* distribution divided group E from the rest of the distribution. Another extensive barrier separated group C from the western range. Within the C region, minor blocks were also present. Further to the west, another block restricted migration between groups SW and W. Regions where gene flow was higher than expected only existed within each geographical zone. All these barriers and corridors were supported by high posterior probability (>0.90) of the migration parameter (Supplemental Figure 2). It should be noted that our sampling focused on representation of major geographical regions of *P. densata*, and extrapolation beyond the sampling range should therefore be interpreted with caution.

Demographic history

To understand the demographic history of the distinct genetic groups in *P. densata*, we plotted the changes in their effective population size (N_e) over time (Figure 2). Group E increased steadily from ~4 Mya, with a slight population contraction at ~1 Mya, leading to a contemporary N_e of ~1.60×10^5. Group C experienced a steep decline at ~0.5 Mya followed by a quick recovery to the previous N_e of 0.81×10^5 and has remained relatively stable since. Group SW experienced a contraction at about 0.4 Mya followed by a recovery and then remained at a stable N_e of 0.32×10^5. Group W had a weak expansion around 0.3 Mya and then maintained a low N_e (~0.23×10^5) over the past 0.3 million years. To validate these results, we used fastsimcoal2 (Excofffier et al., 2021) to establish the most likely demographic scenarios for these groups with multi-population models that consider different modes of gene flow and population size changes. The recovered best-fitting model largely agrees with the Stairway results confirming the bottlenecks in C, with an early contraction at 0.8 Mya in the ancestral population of C and a more recent one at 0.1 Mya. However, the strength of the first bottleneck in C was not as strong as that shown by Stairway, likely owing to differences in accounting for gene flow between the two methods. A detailed presentation of the fastsimcoal results is available in the supplemental information.

Introgression between *P. densata* and *P. yunnanensis*

Detection of introgression using TreeMix suggested that the most likely number of gene flow events (m_{best}) was two, under which the model explained 99.97% of the variation in the data (Supplemental Figure 3). One strong signal of gene flow (29.30%; i.e., the estimated fraction of ancestry in the receiving population derived from the donor) was from *P. yunnanensis* to group SW of *P. densata*, and another weaker signal (12.42%) was between group E and the ancestor of groups SW and W (Figure 3A). ABBA-BABA tests identified eight of the 20 trios with significant *D* values ($p < 0.001$, Supplemental Table 2). By calculating the *f* branch, we assigned significant introgression on the E branch with the other groups of *P. densata* and *P. yunnanensis* (Figure 3B). This gene migration event should be ancient, as contemporary gene flow from E is geographically restricted (Figure 1F). This result agrees with previous studies that recognized the E region as the place of origin of *P. densata* (Song et al., 2002; Wang et al., 2011; Gao et al., 2012; Zhao et al., 2020). The strongest introgression occurred between group SW and *P. yunnanensis* and was also detected by TreeMix. Another gene flow event occurred between group C of *P. densata* and *P. yunnanensis* (Figure 3B). Overall, these results provided evidence of pervasive introgression between *P. yunnanensis* and *P. densata*.

Genomic clines and functional annotation of outliers

The detected introgression between *P. yunnanensis* and *P. densata* led us to explore genomic clines in the species complex to better understand the roles of gene exchange and selection on adaptive evolution. We selected 17 populations along a transition zone that runs from *P. densata* to *P. yunnanensis* to examine patterns of genomic clines. We estimated the degree of admixture and hybrid index for each individual (Figure 4A) and found that hybrid index correlated strongly with elevation (Pearson's $r = -0.9119$, $p < 0.01$, Supplemental Figure 4A). Individuals at lower elevations were genetically more similar to *P. yunnanensis*, whereas those at higher elevations inherited more of their alleles from *P. densata*.

We ran Bayesian estimation of genomic cline (BGC) analysis on 57 849 SNPs in the 17 populations to identify loci with exceptional introgression in the admixed populations. We found 1941 outliers, including 640 positive α, 1227 negative α, 64 positive β, and 14 negative β outliers (Figures 4B and 4C; Supplemental Table 3). The 640 positive α outliers and 1227 negative α outliers represented excess ancestry in *P. yunnanensis* and *P. densata*, respectively. The β parameter represents the rate of introgression, and negative and positive outliers represent loci that may be associated with adaptive introgression and reproductive isolation, respectively. Four of the β outliers were also α outliers.

We annotated the detected outliers to gain a preliminary understanding of their biological significance. Of the 1941 BGC outliers, 684 were located in intergenic regions and the remaining 1257 were in 702 genes (hereafter referred to as BGC-outlier genes, Supplemental Table 3). We classified the 702 BGC-outlier genes into 123 biological categories that were significantly enriched in Gene Ontology (GO) terms (Figure 4D; Supplemental Table 4; $p < 0.05$), including 77 biological process, 32 molecular function, and 14 cellular component terms. In the biological process category, we identified, in addition to growth and material transport and metabolism, a number of terms related to high-elevation adaptation. These included terms related to DNA repair (GO:0033683, GO:0006296, GO:0000731, GO:0006301),

Species	Population	Latitude (°N)	Longitude (°E)	Altitude (m)	*N*	π	Tajima's *D*
P. densata	1 Maerkang	31.91	102.20	2712	8	0.0041	−0.4938
	2 Lixian1	31.67	102.80	2856	8	0.0039	−0.3037
	3 Lixian2	31.40	102.96	2382	8	0.0039	−0.3446
	4 Baoxing	30.78	102.73	2347	8	0.0039	−0.2292
	total group E				32	0.0040	−0.7362
	5 Kangding	30.19	101.91	2951	8	0.0032	−0.2763
	6 Jiulong	29.01	101.50	3025	4	0.0028	−0.0977
	7 Zhongdian1	28.04	99.52	3180	8	0.0029	−0.1609
	8 Zhongdian2	28.18	99.74	3396	8	0.0029	−0.2210
	9 Zhongdian3	27.98	99.71	3547	4	0.0028	−0.1477
	10 Xiangcheng1	28.81	99.86	3804	8	0.0028	−0.1648
	11 Xiangcheng2	28.92	99.79	3234	8	0.0029	−0.2690
	12 Litang	30.52	100.37	2951	8	0.0028	−0.1952
	13 Deqin	28.46	98.86	3242	8	0.0028	−0.1727
	14 Mangkang1	29.20	98.64	3448	8	0.0028	−0.2204
	15 Mangkang2	29.56	98.31	3798	8	0.0028	−0.1979
	total group C				80	0.0029	−0.6613
	16 Zayu1	29.14	97.23	3306	8	0.0025	−0.0088
	17 Zayu2	29.01	97.39	2883	8	0.0025	−0.0622
	18 Zayu3	28.65	97.44	2294	8	0.0025	0.0097
	19 Zayu4	28.48	97.03	1481	8	0.0024	0.0566
	20 Zayu5	28.71	96.79	1991	8	0.0025	0.0231
	total group SW				40	0.0025	−0.1683
	21 Parlung Zangbo1	29.90	95.59	2717	8	0.0021	0.1275
	22 Parlung Zangbo2	30.10	95.10	2152	8	0.0021	0.0525
	23 Niyang valley	29.65	94.38	3220	8	0.0024	0.0484
	24 Yarlung Zangbo	29.25	94.28	2876	8	0.0023	0.0253
	total group W				32	0.0023	−0.0813
	total *P. densata*				184	0.0032	−1.0094
P. yunnanensis	25 Gongshan1	28.02	98.63	1658	8	0.0027	−0.2012
	26 Gongshan2	25.97	98.83	1556	8	0.0026	0.0570
	27 Weixi	27.20	99.29	2262	8	0.0027	−0.0791
	28 Baoshan	24.46	99.14	1936	8	0.003	0.1210
	29 Hutiaoxia	27.23	100.03	1995	8	0.0028	−0.0606
	30 Tengchong	24.92	98.58	1824	8	0.0029	0.0002
	31 Binchuan	25.93	100.41	1898	8	0.0028	0.0399
	32 Lijiang	26.84	100.08	2651	8	0.0027	−0.0969
	33 Wenshan	23.43	104.24	1452	4	0.0027	−0.0928
	total *P. yunnanensis*				68	0.0028	−0.5048
P. tabuliformis	34 Tumote	40.79	111.21	1223	4	0.0039	−0.2444
	35 Guangyuan	32.62	106.10	1340	4	0.0041	−0.1449
	total *P. tabuliformis*				8	0.0041	−0.4188
P. sylvestris	36 northern Sweden				8	0.0042	−0.3181

Table 1. Geographic locations, sample size (*N*), mean nucleotide diversity across all sites (π), and Tajima's *D* of the 36 populations of *P. densata*, *P. tabuliformis*, *P. yunnanensis*, and *P. sylvestris* included in this study.

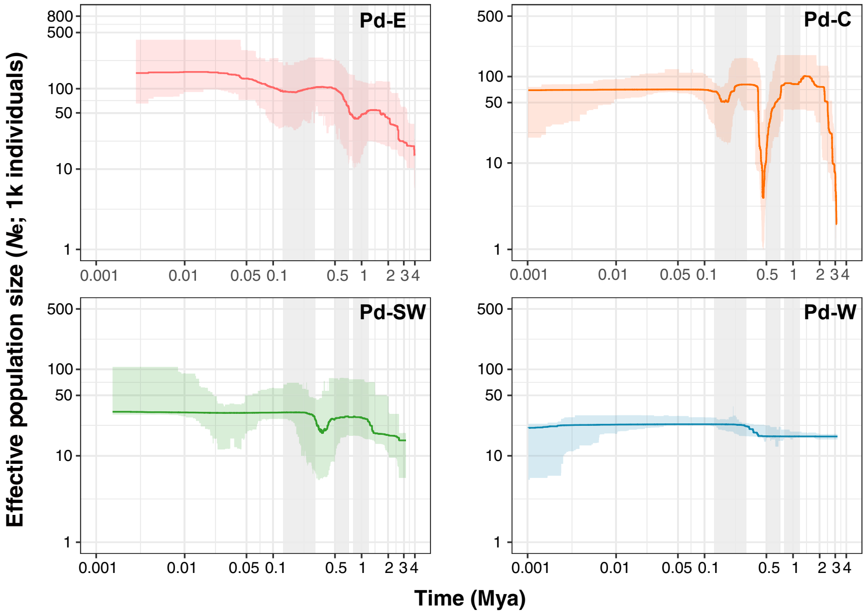

Figure 2. Demographic histories of different groups (E, C, SW, and W) in *P. densata* (Pd) inferred by Stairway Plot 2
Lines represent the median effective population size (N_e) over time, and shaded areas represent 95% confidence intervals. The major glaciations on the QTP, i.e., the Xixiabangma (1.2–0.8 Mya), Naynayxungla (0.7–0.5 Mya), and Guxiang (0.3–0.1 Mya) Glaciations, are highlighted with gray vertical bars.

carbohydrate metabolism (GO:0005351 and GO:0005402), and flavonoid biosynthesis (GO:0009962 and GO:0009963); flavonoids act as sunscreens and antioxidants to prevent damage and/or help plants adapt to a wide range of stress conditions in alpine environments (Baskar et al., 2018).

Kyoto Encyclopedia of Genes and Genomes (KEGG) enrichment analysis revealed six significantly ($p < 0.05$) overrepresented pathways, including ribosome biogenesis in eukaryotes (ko03008), Fanconi anemia pathway (ko03460), circadian rhythm-plant (ko04712), longevity regulating pathway (ko04211), FoxO signaling pathway (ko04068), and glycosaminoglycan degradation (ko00531) (Figure 4E; Supplemental Table 5). The GO and KEGG categorization of genes with extreme introgression patterns provide an overview of the complex molecular mechanisms likely to be involved in high-altitude adaptation in plants.

Association of genomic outliers with environments

We performed redundancy analyses (RDAs) as another test for signatures of genetic adaptation. Forward selection retained three environmental variables (annual mean temperature [bio1], precipitation seasonality [bio15], and wet-day frequency [WET]) and one distance-based Moran's eigenvector map (dbMEM) . Their variance inflation factors (VIFs) were all below 10, suggesting that multicollinearity among these predictors was properly controlled. In the partial RDA that controlled for the spatial component, environment had a large and significant effect ($p = 0.001$, adjusted $R^2 = 26.9\%$) on allele frequencies among populations (Figure 5A). When controlling for environment, the exclusive contribution of spatial distances was not significant ($p = 0.059$, adjusted $R^2 = 1.70\%$; Figure 5A). Partial RDA identified two significant RDA axes ($p < 0.05$) that explained 8.40% and 1.92% of the total variance. The first axis was primarily driven by bio1, whereas the second was associated with bio15 and WET.

RDA identified a total of 387 outlier SNPs significantly correlated with environmental predictors (Figures 5B and 5C; Supplemental Table 3), 102 of which overlapped with the BGC outliers (Figure 5B). Of the 102 RDA-BGC shared outliers, 46 were located in intergenic regions and 56 in 38 genes (Supplemental Table 3). These genes were divided among biological functions influencing growth and development of plants (Supplemental Tables 3 and 4). We examined the association of these 102 loci with environment and found that 61 loci were mostly correlated with bio1, 19 loci with bio15, and 22 loci with WET (Figure 5C; Supplemental Table 3). We selected three loci to illustrate their clinal variation along climate gradients. Locus scaffold653363_26548 is located in a squamosa promoter binding protein-box gene (*SPL8*) that regulates flowering, growth and development, and drought tolerance (Unte et al., 2003; Gou et al., 2018). Allelic distribution of this locus was strongly correlated with bio1 (Figure 5D). Locus tscaffold3033_455200 was annotated as a galactinol synthase gene (*GolS4*) whose encoded enzyme synthesizes galactinol and protects plant cells from oxidative damage (Nishizawa et al., 2008). The allele frequency of this locus co-varied with the precipitation gradient bio15 (Figure 5E). Locus scaffold165922_114751 is located in a gene encoding a member of the cytochrome P450 family (*LUT1*) required for carotenoid epsilon-ring hydroxylation activity (Tian et al., 2004). Allelic frequency distribution at this locus followed the WET gradient (Figure 5F). Interestingly, the hybrid index showed a strong correlation with bio1 ($r^2 = 0.9295$, $p << 0.001$; Figure 6A) and WET ($r^2 = 0.8782$, $p << 0.001$; Figure 6B). When temperature and WET increased, *P. yunnanensis* genotypes increased almost linearly (Figures 6D and 6E).

DISCUSSION

Distinct population structure on the Tibetan Plateau

Wind-pollinated, outcrossing conifers are generally characterized by weak population differentiation over large geographical distances due to extensive gene flow via pollen (Alberto et al., 2013; Hall et al., 2021). Across the large distribution range of *P. tabuliformis* from northern to central China, population differentiation (F_{ST}) in the nuclear genome is only 3% (Xia et al., 2018). By contrast, *P. densata* showed an F_{ST} among groups as high as 18%. Group E of *P. densata* and *P. tabuliformis* shared very similar ancestry in the analyzed genomic regions. Previous studies show that group E is distinguishable from *P. tabuliformis* in mtDNA composition and morphometric traits (Mao et al., 2009; Wang et al., 2011), but the paternally inherited chloroplast DNA is dominated by *P. tabuliformis* types (Wang et al., 2011). This genetic pattern suggests extensive

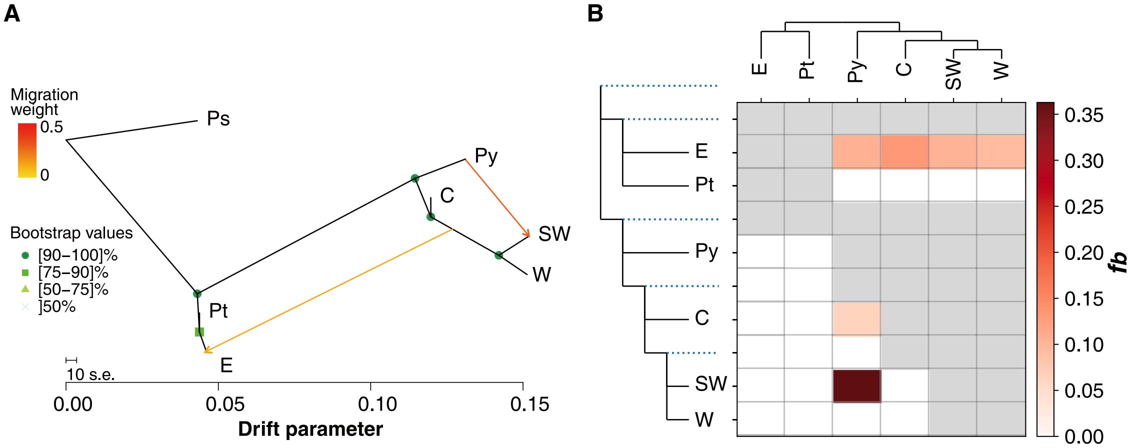

Figure 3. Detection of gene flow and introgression between *P. tabuliformis* (Pt), *P. yunnanensis* (Py), and different groups (E, C, SW, and W) of *P. densata*

(A) Gene flow in the species complex inferred by TreeMix at the most likely migration event (m_{best} = 2). Arrows indicate the direction of gene flow and are colored according to the percentage (weight) of alleles from that source. Scale bars represent a 10-fold average standard error for the entries in the sample covariance matrix. *P. sylvestris* (Ps) was used as an outgroup.

(B) ABBA-BABA tests of introgression based on *D* statistics with Dsuite. The *f*-branch (f_b) statistic identifies possible introgression from the branch of the tree on the y axis to the species/groups on the x axis. Gray cells are empty where comparisons cannot be made.

backcrossing with *P. tabuliformis* via pollen flow in the eastern range of *P. densata* (Wang et al., 2011; Gao et al., 2012) that genetically homogenized the populations of the two species. Whether group E of *P. densata* should be classified as *P. tabuliformis* is an open taxonomic question. Group C showed admixed ancestry from *P. yunnanensis*, but little gene flow was detected in the opposite direction from *P. densata* to *P. yunnanensis*. Asymmetric introgression is commonly observed in hybridizing species complexes (e.g., Arnold et al., 2010; De La Torre et al., 2014; Todesco et al., 2020) and can result from selection on hybrids, genetic incompatibility, or premating barriers between hybridizing populations. We explore these possibilities in the section "Genomic clines associated with adaptation to the alpine environment." The SW and W groups were distinct from each other and from *P. tabuliformis* and *P. yunnanensis*, indicating a long period of isolation from inter- and intraspecific gene exchange. The observed population structure in *P. densata* is consistent with a fragmented migration landscape shaped by high mountains and deep valleys, even over short distances. Furthermore, divergent selection over heterogeneous environments also played a significant role in shaping genetic clines in *P. densata* (Zhao et al., 2020).

High genetic differentiation is a characteristic pattern of mountain biodiversity. We reviewed studies that report nuclear genome-based F_{ST} for plants on QTP and adjacent mountains. Surprisingly, nuclear genome-based studies that report population genetic parameters are scarce among the large number of biogeographical studies of diverse taxonomic groups on the QTP (Wen et al., 2014; Mao et al., 2021). Nevertheless, distinct genetic clusters/lineages within species were commonly detected in the available data (e.g., Fu et al., 2018; Ma et al., 2021; Hu et al., 2022). In *Primula tibetica*, F_{ST} between populations can be as high as 80% (Ren et al., 2017). Populations of a threatened conifer, *Cupressus chengiana*, in the northern Hengduan Mountains have pairwise F_{ST} values of 11%–18% despite its narrow distribution (Li et al., 2020). These cases suggest that habitat isolation together with niche-specific selection promote genetic differentiation and clines, even in conifers capable of extensive gene flow.

Population persistence during glacial periods

Previous studies suggest that the distinct genetic groups of *P. densata* arose through stepwise colonization from E to W during the late Pliocene, as supported by declining population heterozygosity and increasing population differentiation along the migration route (Wang et al., 2011; Gao et al., 2012; Zhao et al., 2020). Reconstructions of the Quaternary climatic history of the QTP recognize four major glaciations: the Xixiabangma, Naynayxungla, Guxiang, and Baiyu (the last) Glaciations, dated at about 1.2–0.8, 0.7–0.5, 0.3–0.1, and 0.07–0.01 Mya, respectively (Zheng et al., 2002). The Naynayxungla Glaciation was the most extensive, covering large areas of the southeastern QTP and forming large ice caps, glacier complexes, and great valley glaciers (Zheng et al., 2002). Our examination of population size changes within each group of *P. densata* correlated well with these regional climate events (Figure 2). The population contraction in group E at about 1 Mya aligned with the Xixiabangma Glaciation (1.2–0.8 Mya). Group C experienced its first strong bottleneck at about 0.5 Mya during the Naynayxungla Glaciation (0.7–0.5 Mya), and another weaker decline at about 0.2 Mya coincided with the Guxiang Glaciation. In contrast to groups E and C, groups SW and W each had 3- to 8-fold smaller effective population sizes and showed little size fluctuation since the mid-Pleistocene. This lack of fluctuation may reflect a smaller impact of glaciaiton on populations that were small from the start than on large, widespread populations . A noticeable pattern in all groups is the rapid population recovery during the interglacial periods after each contraction. This clearly suggests that *P. densata* persisted in multiple regional refugia during the Quaternary ice age and thus preserved the ancient genetic structure that facilitated population rebound.

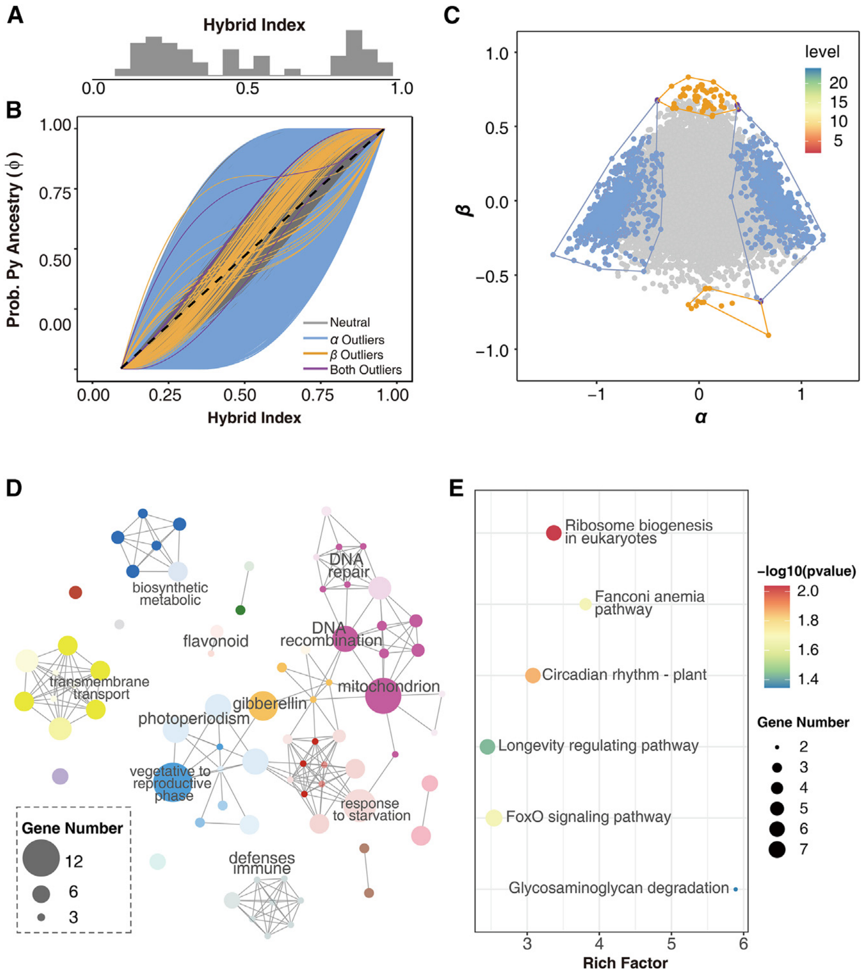

Figure 4. BGC and functional annotation of introgression outlier loci

(A) Histogram depicting the distribution of the hybrid index of the admixed individuals in the contact zone of *P. yunnanensis* (Py) and *P. densata* (Pd). Hybrid index of pure Pd = 0 and pure Py = 1.

(B) Results of BGC, depicting the probability of Py alleles (y axis) given background genomic introgression (i.e., hybrid index; x axis) for 57 849 SNPs. Outlier clines are marked in color, whereas the remainder are gray. The dashed line gives the null expectation based on genome-wide admixture.

(C) An $\alpha \times \beta$ plot illustrates the 2D density of cline width/rate representing the cline center (α; x axis) and steepness of clines (β; y axis). Polygons define density space for significant α (blue), β (orange), and both (purple) outliers.

(D) Network of enriched GO terms of BGC-outlier genes. Node size represents the number of genes annotated to each GO term; each cluster is coded with a different color, and the shading of each node from light to dark represents the *p* value specified by the enrichment test from large to small.

(E) KEGG pathway enrichment for the BGC-outlier genes.

Reconstruction of biogeographical history of diverse plant species frequently reveals the preservation of multiple refugia on the QTP during glacial periods (Opgenoorth et al., 2010; Wen et al., 2014; Ren et al., 2017; Fu et al., 2018; Ma et al., 2021; Hu et al., 2022). The topographic heterogeneity of the QTP and connecting mountains provided rich micro-habitats and broad altitudinal gradients that could have buffered plants from climate fluctuations, allowing their persistence during the Quaternary ice age (Chen et al., 2019; Ding et al., 2020; Guo et al., 2022). The preservation of scattered refugial populations may have led to rapid local range expansion during the post- or interglacial periods and thus to deep intraspecific divergence. Such population dynamics likely underlay the evolution of high biodiversity in the QTP and other mountain systems.

Genomic clines associated with adaptation to the alpine environment

Zones of hybridization and introgression are good settings for understanding genetic differences that are potentially associated with adaptation and reproductive isolation between diverging species (Barton and Hewitt, 1989; Buerkle and Lexer, 2008). We selected a transition zone between *P. densata* and *P. yunnanensis* for genomic cline analyses for two reasons: (1) the populations in this zone are distributed along an elevation gradient that runs north to south, with a contact zone and admixed populations in the middle; (2) they are distributed along major river valleys that provide corridors for gene flow. Thus, possibilities for gene exchange among populations along valleys are not hindered by major geographical barriers, and an isolation-by-distance model provides a valid neutral expectation.

BGC identified exceptional introgression at 3.36% (1941) of the 57 849 loci analyzed in the admixed populations. Approximately two-thirds of these outlier loci showed excess *P. densata* ancestry and one-third showed excess *P. yunnanensis* ancestry. This could be the result of either divergent selection operating on different loci within the contact zone or genetic drift. Among the 1941 BGC outlier loci, 14 showed more rapid than expected introgression rates (negative β), suggestive of adaptive introgression, and 64 showed slower than expected introgression (positive β), suggestive of loci associated with reproductive barriers (Gompert and Buerkle, 2012). In the genus *Pinus*, widespread intrinsic genetic incompatibilities are absent among closely related taxa (Critchfield, 1975). We previously examined the genetic compatibility between *P. densata*, *P. yunnanensis*, and *P. tabuliformis* in a controlled crossing experiment and found that the crossability between *P. densata* and *P. yunnanensis* was as high as 0.51 (Zhao et al., 2014). Thus, detection of numerous outliers associated with intrinsic reproductive isolation is not expected among crossable pines. In the genic view of speciation, reproductive isolation can be achieved by a small number of major barrier loci (Wu, 2001). The potential roles of these 64 outliers in reproductive isolation should therefore be investigated further.

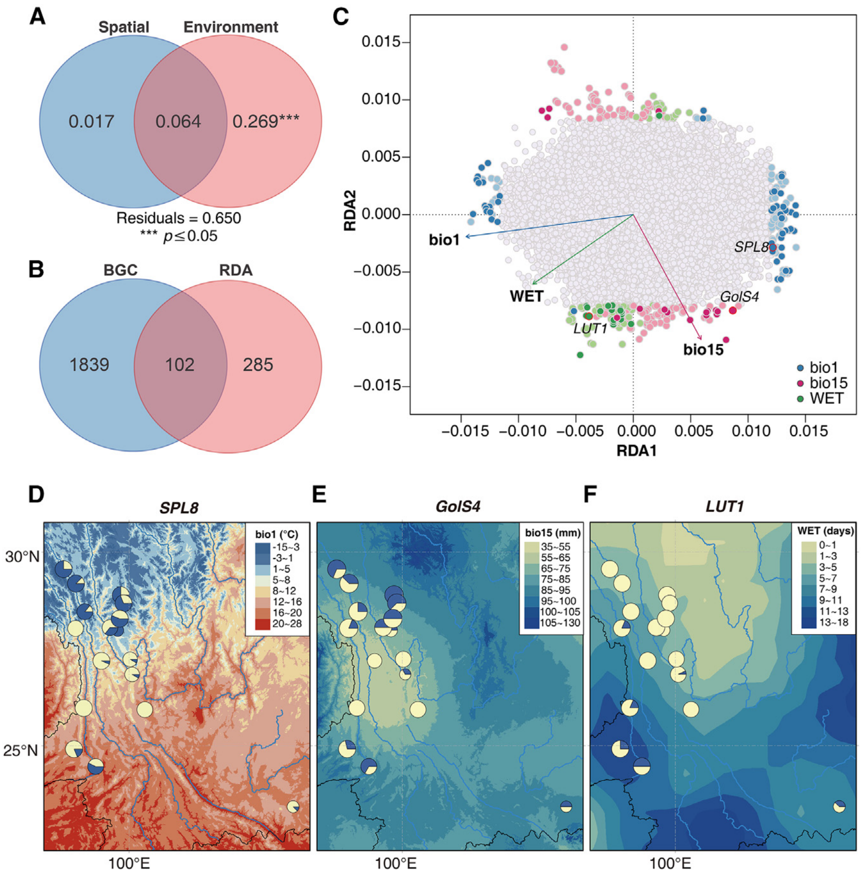

Figure 5. RDA and identification of loci associated with environmental variables

(A) Partial RDAs that estimate the effects of spatial vs. environmental predictors on population allele frequencies.

(B) Venn diagram depicting overlap between outliers identified by BGC and RDA.

(C) Significant environment associated outliers identified by RDA, with the best-supported SNP–environment association in the same color. Dark colors represent the 102 RDA–BGC outliers.

(D–F) Illustration of allelic frequency variation along climate gradients of three outlier loci located on genes *SPL8*, *GolS4*, and *LUT1* and their association with bio1, bio15, and WET, respectively. The frequencies of the two alternative alleles at each locus are shown in pie charts.

It has been suggested that, when hybrid fitness involves many loci with different impacts, the loci undergoing the strongest selection will likely be labeled as β outliers, whereas weakly selected loci will be shown as having excess ancestry (Gompert and Buerkle, 2012). This is likely to be the case in a hybrid zone between the two North American pine species *Pinus strobiformis* and *Pinus flexilis*, where thousands of excess-ancestry outliers but no β outliers are found (Menon et al., 2018). This pattern of genomic cline is regarded as supporting evidence for species boundaries that are maintained by external ecological factors (Ryan et al., 2017; Menon et al., 2018). Simulations also indicate that although both genetic drift and selection can cause excess ancestry and variable introgression, loci with excess ancestry are often enriched in selected loci (Gompert and Buerkle, 2012; Gompert et al., 2012). Most studies of conifer hybrid zones have recognized that ecological selection plays a dominant role as a driver of species diversification, but intrinsic isolation loci are rarely recovered (Holliday et al., 2010; De La Torre et al., 2014; Menon et al., 2018). Our results thus suggest that intrinsic genetic barriers may evolve faster when selection pressure is high, and this is likely the case in harsh or contrasting habitats. This explains differences in the recovery of introgression outliers among studies because the results reflect specific population–environment dynamics.

Our detection of strong clines in the outliers, their association with critical climatic factors, and the significant impact of environment on population divergence substantiate selection along climate gradients as a driving force for differential introgression, and thus the delineation of species distributions. The enrichment of genes related to adaptation to the alpine environment in the outlier loci adds functional support for the biological significance of the outliers. Although it is speculative to link these signals to local adaptation without molecular and functional validation, they nevertheless constitute a coherent set of evidence for a role of ecological selection in facilitating adaptive divergence in alpine environments.

Overall, our study highlights the contributions of introgression, migration history, and barriers to gene flow over fragmented and heterogeneous landscapes to the broad-scale population structure and genetic clines of *P. densata*. Climatic fluctuations in the Quaternary caused recurrent cycles of population decline and post-glacial expansion, a process that is characteristic of the biogeographical history of QTP flora. The preservation of ancient genetic lineages and divergent selection over heterogeneous habitats are important drivers of intra- and interspecific diversification in this important biodiversity hotspot. The association of genomic clines with environmental clines also provided valuable perspectives on species boundary dynamics. We uncovered genomic imprints of ecological adaptation and reproductive isolation across a species transition zone, suggesting that both intrinsic genetic barriers and extrinsic ecological selection play a key role in maintaining species boundaries. Thus, although gene flow tends to homogenize connected populations, the coupling of genetic and ecological factors could increase reproductive isolation and ensure species separation and divergence. However, much remains to be done to relate genomic patterns to evolutionary processes. Whole-genome resequencing and better annotation of the pine genome will continue to refine our understanding of the targets, tempo, and modes of selection in this species complex and conifer evolution in general.

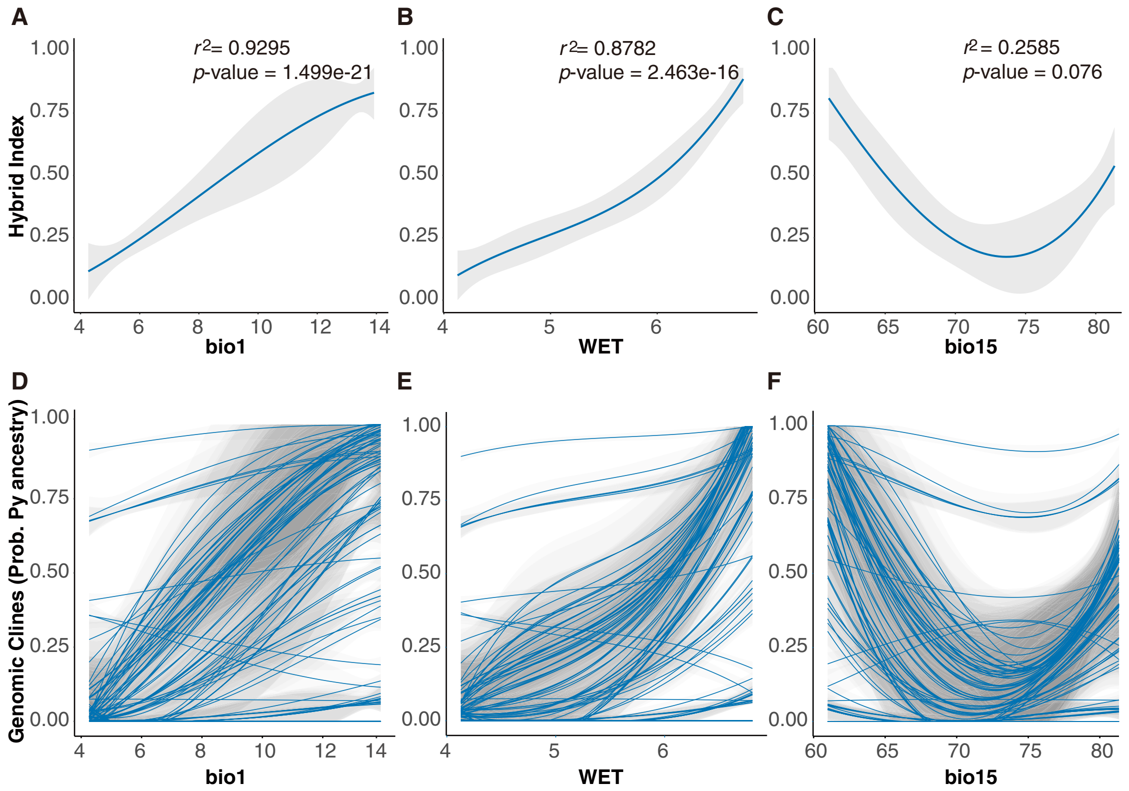

Figure 6. Correlation of environmental variables with genomic clines

(A–F) Hybrid index of admixed individuals vs. bio1 **(A)**, WET **(B)**, and bio15 **(C)**. The genomic clines, shown as fitted values for the reference population *P. yunnanensis* (Py) homozygote for each locus and individual, of the 102 BGC–RDA outliers vs. environmental variables bio1 **(D)**, WET **(E)**, and bio15 **(F)**. The gray shading indicates 95% confidence intervals for each regression line.

MATERIALS AND METHODS

Population sampling and exome capture sequencing

This study included 24 populations of *P. densata*, two representative populations of *P. tabuliformis*, and nine populations of *P. yunnanensis* (Figure 1A). Each population included eight individuals, except for five populations from which four individuals were collected. The sampled trees in each local stand were separated by at least 50 m from each other. Identification and sampling of *P. densata* were guided by species distribution maps, morphology, and mtDNA composition. Populations from the eastern margin of *P. densata* (group E in this study) exhibit heterogeneous maternal lineages as shown by a mix of mtDNA haplotypes found in the two parent species (Wang et al., 2011). Phenotypically, these eastern populations are distinct from the two parental species in cone and seed morphometric traits (Mao et al., 2009). The name, location, and sample size of each population are listed in Table 1. Of the 36 populations, 27 (nos. 1–6, 8–25, and 33–35) have been used for exome capture sequencing in a previous study (Zhao et al., 2020). The other nine populations (nos. 7, 26–32, 36) were new collections for this study. One population of *P. sylvestris* was included as an outgroup.

Needles were collected from individual trees in each natural stand, and genomic DNA was extracted using a Plant Genomic DNA Kit (Tiangen, Beijing, China). We performed probe capture sequencing using the same set of 40 000 exome probes as in Zhao et al. (2020). The probes, each 120 nt in length, were designed from *P. taeda* UniGenes (Neves et al., 2013). The majority of the probes aligned to c. 29 000 genes, and 9800 probes aligned to intergenic regions. The laboratory work consisted of three major steps: (1) genomic DNA fragmentation, (2) hybridization to capture probes, and (3) paired-end sequencing (2× 101 bp) of the captured DNA fragments on the HiSeq 2000 platform (Illumina) (Neves et al., 2013). Library preparation, probe hybridization, and sequencing were conducted by RAPiD Genomics (Gainesville, FL, USA). In total, 268 samples were successfully genotyped and analyzed in this study.

Bioinformatics

Sequence read quality was assessed with FastQC (http://www.bioinformatics.babraham.ac.uk/projects/fastqc/). Adapter sequences and low-quality bases (Phred quality <20) were removed using Trimmomatic (Bolger et al., 2014). Reads shorter than 36 bases after trimming were discarded. Clean reads were mapped to the *P. taeda* v.1.01 assembly (Neale et al., 2014) using the BWA-MEM algorithm with default parameters (Li, 2013) to produce BAMs.

To reduce the computation time of the Genome Analysis Toolkit (GATK) pipeline (Van der Auwera et al., 2013), we prepared reduced BAM files and the corresponding reduced reference for each sample. In brief, variants were called with the SAMtools and BCFtools pipeline using default parameters (Li, 2011) for all 268 samples. Scaffolds that had at least one SNP in ≥50% of the individuals were included in the reduced reference. The reduced BAMs were prepared with SAMtools based on the new reduced reference. PCR duplicates were removed using Picard MarkDuplicates (http://broadinstitute.github.io/picard/). Reads around indels were locally realigned using RealignerTargetCreator and IndelRealigner in GATK. Variant calling was performed using HaplotypeCaller. GenotypeGVCFs was then used to perform multisample

joint aggregation and genotype likelihood correction with the parameter "includeNonVariantSites."

Several filtering steps were performed to minimize genotyping errors: indels were removed, SNPs located in repetitive regions (with reference to the *P. taeda* genome v.1.01) and with mapping quality <40 were removed, genotypes with genotype quality <20 or read depth <3 were masked as missing, and SNPs that met any of the following criteria were removed: missing rate >20%, MAF < 5%, heterozygosity >70%, or allele number >2. The remaining SNPs were used for population genomic analyses, with the exception of site frequency spectrum (SFS) and nucleotide diversity-based analyses, for which no MAF filtering was applied.

Population structure and diversity

Population genetic structure was examined using FastSTRUCTURE (Raj et al., 2014), with the number of genetic clusters (*K*) required to explain the structure in the dataset set to 1–16. The best *K* value that maximized the marginal likelihood of clustering in the data was identified by the script "chooseK.py". The graphical illustration of ancestry coefficients was plotted using "distruct.py". Differentiation (F_{ST}) between genetic clusters was estimated using the R package HIERFSTAT (Goudet, 2005). Population structure was also investigated using PCA implemented in EIGENSOFT v6.1.4 (Price et al., 2006). An NJ tree among the samples was constructed using PHYLIP version 3.698 (Felsenstein, 2005). Nucleotide diversity (π) (Nei and Li, 1979) and Tajima's *D* (Tajima, 1989) of each population and group were calculated including both informative and invariant sites with VCFtools 0.1.16 (Danecek et al., 2011).

Estimated effective migration surface

To visualize spatial patterns of gene flow, we used EEMS (Petkova et al., 2016). EEMS uses a stepping-stone model (Kimura and Weiss, 1964) to identify geographic regions where decays in genetic similarity deviate from isolation-by-distance predictions, thus highlighting dispersal corridors or barriers. EEMS was run using a deme size of 800 with a Markov chain Monte Carlo (MCMC) length of 20 million iterations following a burn-in of 10 million and a thinning interval of 9999. The reliability of EEMS models was evaluated by checking the linear relationship between the observed and fitted dissimilarities within and between demes. Finally, the surfaces of effective migration rates (*m*) were generated and plotted using the rEEMSplot package (https://github.com/dipetkov/eems).

Inference of demographic history

Population genetic analyses identified four distinct groups in *P. densata* (see Results). We inferred the demographic histories of these groups (E, C, W, and SW) using the flexible multi-epoch coalescent approach implemented in Stairway Plot 2 (Liu and Fu, 2020). We constructed the folded one-dimensional SFS for each species or group by performing the down projection method using the Python script "easySFS" (https://github.com/isaacovercast/easySFS). The final folded SFS for *P. densata* contained 7 544 066 sites. We set the mutation rate to 7×10^{-10} per site per year and the generation time to 50 years (Willyard et al., 2007). Two hundred subsamples of 67% of all sites were generated to estimate the median and 95% confidence interval of effective population size (*N*e) over time. We also used another coalescent simulation-based method, fastsimcoal2 version 2.7 (Excofffier et al., 2021), to infer the demographic history of *P. densata* groups. We tested 11 scenarios, which differed in (1) the levels of gene flow between groups, (2) the mode of population size change after splitting from the ancestral population, and (3) whether and when strong bottlenecks occurred. Detailed descriptions of fastsimcoal model testing are presented in the supplemental information.

Detecting introgression

To infer the potential presence of gene flow between distinct genetic groups, we applied a composite-likelihood approach implemented in TreeMix v1.12 (Pickrell and Pritchard, 2012) on linkage disequilibrium (LD)-pruned data. We identified and removed variants in strong LD (r^2 threshold of 0.05) along windows of 50 SNPs and a step size of five SNPs using PLINK (Purcell et al., 2007; Chang et al., 2015). We tested the addition of 0–10 migration events by building 100 replicate maximum likelihood trees for each migration event. Potential migration events were inferred when the proportion of explained covariance among groups stabilized toward their maximum asymptotic values. These analyses were implemented in the BITE R package (Milanesi et al., 2017).

We also performed ABBA-BABA (also known as Patterson's *D*) statistical tests with Dsuite version 0.4r43 to detect introgression (Malinsky et al., 2021). The ABBA-BABA test involves four populations or taxa in the form (((P1, P2), P3), outgroup) and determines potential gene flow between P3 and P1 or P2 based on the relative site patterns of ABBA and BABA. We inferred phylogenetic relationships among the populations by SVDquartets (Chifman and Kubatko, 2014) using the same set of SNPs as in TreeMix, and we uploaded the recovered tree to Dsuite. A total of 20 trios of (((P1, P2), P3), *P. sylvestris*) were tested (Supplemental Table 2). The significance of each test was assessed using 100 jackknife resampling runs. Branch-specific gene flow was estimated based on the f_4 ratio using the Dtrios and Fbranch program in Dsuite.

Analysis of genomic clines

ABBA-BABA tests detected introgression between *P. yunnanensis* and group C of *P. densata* (see Results). We selected eight populations from *P. densata* group C and nine populations of *P. yunnanensis* that formed a transition from pure *P. yunnanensis* to admixed populations with *P. densata* to pure *P. densata*. Additionally, they also formed a geographical gradient along the major river valleys of the region that run north to south following elevations (Supplemental Figure S4B). We investigated patterns of differential introgression between *P. yunnanensis* and *P. densata* using BGCs (Gompert and Buerkle, 2012). BGC identifies introgression by quantifying the probability of locus-specific ancestry given genome-wide ancestry or hybrid index (Gompert and Buerkle, 2012). The hybrid index is the proportion φ of an individual's genome inherited from reference population 1 (P1); the probability of being inherited from reference population 2 (P2) is therefore $1 - \varphi$. BGC summarizes introgression using a locus-specific α that quantifies the change in ancestry relative to a null expectation based on the genome-wide hybrid index and a β that specifies the slope of the cline (Gompert and Buerkle, 2012). Loci identified as α outliers may be involved in directional selection toward homozygous genotypes from one reference population or selection against heterozygotes (Gompert and Buerkle, 2011; Oswald et al., 2019). Negative β values indicate faster-than-expected introgression rates, whereas positive β values denote rates slower than expected. Loci that are identified as β outliers may be involved in reproductive isolation (positive β) or adaptive introgression (negative β) (Gompert and Buerkle, 2012). For both parameters, a zero value corresponds to the null model.

We classified our sampled trees into admixed or reference genotypes based on the Q-values of ADMIXTURE (Alexander et al., 2009). We considered a population to be admixed when its inferred ancestry coefficient (mean Q value) was between 0.1 and 0.9, and those with Q < 0.1 or Q > 0.9 were considered to be from the two reference populations. Under these criteria, we identified seven pure *P. yunnanensis* populations (P1), four pure *P. densata* populations (P2), and six admixed populations (Supplemental Figure S4C). We ran BGC three times independently for 400 000 MCMC iterations each time with a burn-in of 200 000 and a thinning interval of 100, and we assessed convergence by visual inspection (Supplemental Figures 4D–4F). Replicate runs were subsequently combined. BGC outliers were identified as credible when their 95% confidence intervals excluded the neutral expectation (i.e., α or β = 0) or their median of posterior distribution surpassed the $(1-0.975)/2$ and 0.975/2 quantiles of

the probability distribution (Gompert and Buerkle, 2012). Genomic clines were visualized using the ClineHelpR R package (Martin et al., 2021).

Genotype–environment association

To examine the role of environmental selection on genomic clines in the contact zone, we performed RDAs to identify outlier loci associated with environmental variables in the 17 populations analyzed for BGC. RDA is a multivariate constrained ordination approach that combines multiple linear regression and PCA to detect significant loci associated with environmental gradients (Legendre and Legendre, 2012).

We included the Hellinger-transformed allele frequencies of each population as the response matrix and two predictor matrices of environmental and spatial variables, respectively. We extracted 12 environmental variables for each sampling site (Supplemental Table 6): annual mean air temperature (bio1), isothermality (bio3), air temperature seasonality (bio4), annual precipitation (bio12), precipitation of the driest month (bio14), precipitation seasonality (bio15), ground-frost frequency (FRS), growing degree days, soil organic carbon, wet-day frequency (WET), annual mean UV-B (UVB1), and elevation (ELEV). These variables have previously been identified as most relevant to niche divergence in *P. densata* and other pine species (Mao and Wang, 2011; Zhao et al., 2020). All raster layers were converted to the same resolution (30 arc-seconds). To account for underlying spatial influences, we used the dbMEMs as spatial predictors. dbMEMs are orthogonal spatially explicit eigenvectors that capture spatial patterns from multiple angles rather than from simple latitudinal and longitudinal vectors. To minimize model overfitting, forward selection with 1000 permutations was performed with an α value of 0.05 on the spatial and environmental variables separately (Borcard et al., 2011).

A partial RDA conditioned on the dbMEM spatial matrix was then performed to identify adaptive loci with allele frequencies as the response variable and the environment matrix as the predictor variable using the vegan R package (Oksanen et al., 2019). Significant RDA axes were tested using the anova.cca function with a permutation test of 9999 iterations. VIFs for the predictor variables used in the model were evaluated using the vif.caa function. We extracted the SNP loadings from each of the significant RDA axes and chose three standard deviations (two-tailed $p = 0.0027$) as the cutoff for outliers. Each SNP was then associated with all environmental predictors using Pearson's correlation coefficient (r) (Forester et al., 2018), and those with the strongest correlations were regarded as the best-supported SNP–environment associations. Similarly, we performed another partial RDA conditioned on environment to estimate the effects of spatial vs. environmental predictors.

Outlier annotation and enrichment analyses

Outlier SNPs were annotated and categorized using SnpEff v4.3t (Cingolani et al., 2012) on the basis of their impact, functional class, and genomic region. The functional annotations of all protein-coding genes in the *P. taeda* v1.01 assembly were determined using eggNOG-mapper v2 (Cantalapiedra et al., 2021) with parameters -m diamond –tax_scope 33090. We extracted the GO terms and KEGG pathways from the eggNOG-mapper results to build a Pita Orgdb database using the AnnotationForge R package v1.30.1. Based on this Orgdb database, GO and KEGG enrichment analyses were performed in the clusterProfiler R package v3.16.1. We used GOMCL (Wang et al., 2020) to reduce redundancy and summarize the lists of GO terms and displayed the results with Cytoscape 3.8.2 (Shannon et al., 2003).

DATA AVAILABILITY

All sequencing data are archived in the NCBI SRA database (BioProjects PRJNA891676 and PRJNA492187) and the China National Genomics Data Center (BioProject PRJCA015270). The VCF genotype files of the samples are deposited at Dryad: https://doi.org/10.5061/dryad.sxksn0373.

SUPPLEMENTAL INFORMATION

Supplemental information is available at *Plant Communications Online*.

FUNDING

This study was supported by grants from the Swedish Research Council (VR 2017-04686) and T4F program, Sweden.

AUTHOR CONTRIBUTIONS

X.-R.W. and W.Z. designed the study. W.Z. and J.-F.M. conducted field sampling. J.-F.G., W.Z., and B.A. analyzed the data. J.-F.G., W.Z., and X.-R.W. wrote the manuscript draft. All authors contributed to the revision of the manuscript.

ACKNOWLEDGMENTS

We thank Prof. Barbara Giles for valuable comments and linguistic editing that improved the clarity of the manuscript. Genomic data processing and analyses were performed using resources provided by the Swedish National Infrastructure for Computing (SNIC) through the High Performance Computing Centre North (HPC2N). No conflict of interest is declared.

Received: October 18, 2022
Revised: February 9, 2023
Accepted: March 8, 2023
Published: March 11, 2023

REFERENCES

Alberto, F.J., Aitken, S.N., Alía, R., et al. (2013). Potential for evolutionary responses to climate change - evidence from tree populations. Glob. Chang. Biol. **19**:1645–1661. https://doi.org/10.1111/gcb.12181.

Alexander, D.H., Novembre, J., and Lange, K. (2009). Fast model-based estimation of ancestry in unrelated individuals. Genome Res. **19**:1655–1664. https://doi.org/10.1101/gr.094052.109.

Arnold, M.L., Tang, S., Knapp, S.J., and Martin, N.H. (2010). Asymmetric introgressive hybridization among Louisiana Iris species. Genes **1**:9–22. https://doi.org/10.3390/genes1010009.

Barton, N.H. (2001). The role of hybridization in evolution. Mol. Ecol. **10**:551–568. https://doi.org/10.1046/j.1365-294x.2001.01216.x.

Barton, N.H., and Hewitt, G.M. (1989). Adaptation, speciation and hybrid zones. Nature **341**:497–503. https://doi.org/10.1038/341497a0.

Baskar, V., Venkatesh, R., and Ramalingam, S. (2018). Flavonoids (antioxidants systems) in higher plants and their response to stresses. In Antioxidants and Antioxidant Enzymes in Higher Plants, D.K. Gupta, J.M. Palma, and F.J. Corpas, eds. (Cham: Springer International Publishing), pp. 253–268. https://doi.org/10.1007/978-3-319-75088-0_12.

Bolger, A.M., Lohse, M., and Usadel, B. (2014). Trimmomatic: a flexible trimmer for Illumina sequence data. Bioinformatics **30**:2114–2120. https://doi.org/10.1093/bioinformatics/btu170.

Borcard, D., Gillet, F., and Legendre, P. (2011). Numerical Ecology with R (Springer International Publishing).

Buerkle, C.A., and Lexer, C. (2008). Admixture as the basis for genetic mapping. Trends Ecol. Evol. **23**:686–694. https://doi.org/10.1016/j.tree.2008.07.008.

Cantalapiedra, C.P., Hernández-Plaza, A., Letunic, I., Bork, P., and Huerta-Cepas, J. (2021). eggNOG-mapper v2: functional annotation, orthology assignments, and domain prediction at the metagenomic scale. Mol. Biol. Evol. **38**:5825–5829. https://doi.org/10.1093/molbev/msab293.

Chang, C.C., Chow, C.C., Tellier, L.C., Vattikuti, S., Purcell, S.M., and Lee, J.J. (2015). Second-generation PLINK: rising to the challenge of larger and richer datasets. GigaScience **4**:7. https://doi.org/10.1186/s13742-015-0047-8.

Chen, J.H., Huang, Y., Brachi, B., Yun, Q.Z., Zhang, W., Lu, W., Li, H.N., Li, W.Q., Sun, X.D., Wang, G.Y., et al. (2019). Genome-wide analysis of Cushion willow provides insights into alpine plant divergence in a biodiversity hotspot. Nat. Commun. **10**:5230. https://doi.org/10.1038/s41467-019-13128-y.

Cheng, W.C., and Fu, L.G. (1978). Chinese Flora (Science Press).

Chifman, J., and Kubatko, L. (2014). Quartet inference from SNP data under the coalescent model. Bioinformatics **30**:3317–3324. https://doi.org/10.1093/bioinformatics/btu530.

Cingolani, P., Platts, A., Wang, L.L., Coon, M., Nguyen, T., Wang, L., Land, S.J., Lu, X., and Ruden, D.M. (2012). A program for annotating and predicting the effects of single nucleotide polymorphisms, SnpEff: SNPs in the genome of *Drosophila melanogaster* strain w^{1118}; *iso*-2; *iso*-3. Fly **6**:80–92. https://doi.org/10.4161/fly.19695.

Colicchio, J.M., Hamm, L.N., Verdonk, H.E., Kooyers, N.J., and Blackman, B.K. (2021). Adaptive and nonadaptive causes of heterogeneity in genetic differentiation across the *Mimulus guttatus* genome. Mol. Ecol. **30**:6486–6507. https://doi.org/10.1111/mec.16087.

Critchfield, W.B. (1975). Interspecific hybridization in *Pinus*: a summary review. In Symp. On Interspecific and Interprovenance Hybridization in Forest Trees, D.P. Fowler and C.Y. Yeatman, eds.. Proc. 14th Meeting, Canad (Tree Improv. Assoc., Part II), pp. 99–105.

Cruickshank, T.E., and Hahn, M.W. (2014). Reanalysis suggests that genomic islands of speciation are due to reduced diversity, not reduced gene flow. Mol. Ecol. **23**:3133–3157. https://doi.org/10.1111/mec.12796.

Danecek, P., Auton, A., Abecasis, G., Albers, C.A., Banks, E., DePristo, M.A., Handsaker, R.E., Lunter, G., Marth, G.T., Sherry, S.T., et al. (2011). The variant call format and VCFtools. Bioinformatics **27**:2156–2158. https://doi.org/10.1093/bioinformatics/btr330.

De La Torre, A.R., Wang, T., Jaquish, B., and Aitken, S.N. (2014). Adaptation and exogenous selection in a *Picea glauca* x *Picea engelmannii* hybrid zone: implications for forest management under climate change. New Phytol. **201**:687–699. https://doi.org/10.1111/nph.12540.

Ding, W.N., Ree, R.H., Spicer, R.A., and Xing, Y.W. (2020). Ancient orogenic and monsoon-driven assembly of the world's richest temperate alpine flora. Science **369**:578–581. https://doi.org/10.1126/science.abb4484.

Excofffier, L., Marchi, N., Marques, D.A., Matthey-Doret, R., Gouy, A., and Sousa, V.C. (2021). fastsimcoal2: demographic inference under complex evolutionary scenarios. Bioinformatics **37**:4882–4885. https://doi.org/10.1093/bioinformatics/btab468.

Felsenstein, J. (2005). PHYLIP (Phylogeny Inference Package) Version 3.6. Distributed by the Author (Seattle: Department of Genome Sciences, University of Washington).

Fitzpatrick, B.M. (2013). Alternative forms for genomic clines. Ecol. Evol. **3**:1951–1966. https://doi.org/10.1002/ece3.609.

Forester, B.R., Lasky, J.R., Wagner, H.H., and Urban, D.L. (2018). Comparing methods for detecting multilocus adaptation with multivariate genotype-environment associations. Mol. Ecol. **27**:2215–2233. https://doi.org/10.1111/mec.14584.

Fu, P.C., Ya, H.Y., Liu, Q.W., Cai, H.M., and Chen, S.L. (2018). Out of refugia: population genetic structure and evolutionary history of the alpine medicinal pant *Gtiana lawrencei* var. *farreri* (Gentianaceae). Front. Genet. **9**:564. https://doi.org/10.3389/fgene.2018.00564.

Gao, J., Wang, B., Mao, J.F., Ingvarsson, P., Zeng, Q.Y., and Wang, X.R. (2012). Demography and speciation history of the homoploid hybrid pine *Pinus densata* on the Tibetan Plateau. Mol. Ecol. **21**:4811–4827. https://doi.org/10.1111/j.1365-294X.2012.05712.x.

Gompert, Z., and Buerkle, C.A. (2011). Bayesian estimation of genomic clines. Mol. Ecol. **20**:2111–2127. https://doi.org/10.1111/j.1365-294X.2011.05074.x.

Gompert, Z., and Buerkle, C.A. (2012). bgc: software for Bayesian estimation of genomic clines. Mol. Ecol. Resour. **12**:1168–1176. https://doi.org/10.1111/1755-0998.12009.x.

Gompert, Z., Parchman, T.L., and Buerkle, C.A. (2012). Genomics of isolation in hybrids. Philos. Trans. R. Soc. Lond. B Biol. Sci. **367**:439–450. https://doi.org/10.1098/rstb.2011.0196.

Gou, J., Debnath, S., Sun, L., Flanagan, A., Tang, Y., Jiang, Q., Wen, J., and Wang, Z.Y. (2018). From model to crop: functional characterization of *SPL8* in *M. truncatula* led to genetic improvement of biomass yield and abiotic stress tolerance in alfalfa. Plant Biotechnol. J. **16**:951–962. https://doi.org/10.1111/pbi.12841.

Goudet, J. (2005). Hierfstat, a package for r to compute and test hierarchical *F*-statistics. Mol. Ecol. Notes **5**:184–186. https://doi.org/10.1111/j.1471-8278.

Guo, J., Wang, B., Liu, Z., Mao, J., Wang, X., and Zhao, W. (2022). Low genetic diversity and population connectivity fuel vulnerability to climate change for the Tertiary relict pine *Pinus bungeana*. J. Systemat. Evol. **61**:143–156. https://doi.org/10.1111/jse.12821.

Hall, D., Olsson, J., Zhao, W., Kroon, J., Wennström, U., and Wang, X.R. (2021). Divergent patterns between phenotypic and genetic variation in Scots pine. Plant Commun. **2**, 100139. https://doi.org/10.1016/j.xplc.2020.100139.

Hamilton, J.A., Lexer, C., and Aitken, S.N. (2013). Genomic and phenotypic architecture of a spruce hybrid zone (*Picea sitchensis* × *P. glauca*). Mol. Ecol. **22**:827–841. https://doi.org/10.1111/mec.12007.

Holliday, J.A., Ritland, K., and Aitken, S.N. (2010). Widespread, ecologically relevant genetic markers developed from association mapping of climate-related traits in Sitka spruce (*Picea sitchensis*). New Phytol. **188**:501–514. https://doi.org/10.1111/j.1469-8137.2010.03380.x.

Hu, H., Yang, Y., Li, A., Zheng, Z., Zhang, J., and Liu, J. (2022). Genomic divergence of *Stellera chamaejasme* through local selection across the Qinghai-Tibet plateau and northern China. Mol. Ecol. **31**:4782–4796. https://doi.org/10.1111/mec.16622.

Jin, W.T., Gernandt, D.S., Wehenkel, C., Xia, X.M., Wei, X.X., and Wang, X.Q. (2021). Phylogenomic and ecological analyses reveal the spatiotemporal evolution of global pines. Proc. Natl. Acad. Sci. USA **118**, e2022302118. https://doi.org/10.1073/pnas.2022302118.

Kimura, M., and Weiss, G.H. (1964). The stepping stone model of population structure and the decrease of genetic correlation with distance. Genetics **49**:561–576. https://doi.org/10.1093/genetics/49.4.561.

Legendre, P., and Legendre, L. (2012). Numerical Ecology (Elsevier).

Li, H. (2011). A statistical framework for SNP calling, mutation discovery, association mapping and population genetical parameter estimation from sequencing data. Bioinformatics **27**:2987–2993. https://doi.org/10.1093/bioinformatics/btr509.

Li, H. (2013). Aligning Sequence Reads, Clone Sequences and Assembly Contigs with BWA-MEM. Preprint at arXiv. https://doi.org/10.48550/arXiv.1303.3997.

Li, J., Milne, R.I., Ru, D., Miao, J., Tao, W., Zhang, L., Xu, J., Liu, J., and Mao, K. (2020). Allopatric divergence and hybridization within *Cupressus chengiana* (Cupressaceae), a threatened conifer in the northern Hengduan Mountains of western China. Mol. Ecol. **29**:1250–1266. https://doi.org/10.1111/mec.15407.

Liu, X., and Fu, Y.X. (2020). Stairway Plot 2: demographic history inference with folded SNP frequency spectra. Genome Biol. **21**:280. https://doi.org/10.1186/s13059-020-02196-9.

Ma, Y.P., Wariss, H.M., Liao, R.L., Zhang, R.G., Yun, Q.Z., Olmstead, R.G., Chau, J.H., Milne, R.I., Van de Peer, Y., and Sun, W.B. (2021). Genome-wide analysis of butterfly bush (*Buddleja alternifolia*) in three uplands provides insights into biogeography, demography and speciation. New Phytol. **232**:1463–1476. https://doi.org/10.1111/nph.17637.

Malinsky, M., Matschiner, M., and Svardal, H. (2021). Dsuite - fast *D*-statistics and related admixture evidence from VCF files. Mol. Ecol. Resour. **21**:584–595. https://doi.org/10.1111/1755-0998.13265.

Mao, J.F., and Wang, X.R. (2011). Distinct niche divergence characterizes the homoploid hybrid speciation of *Pinus densata* on the Tibetan Plateau. Am. Nat. **177**:424–439. https://doi.org/10.1086/658905.

Mao, J.F., Li, Y., and Wang, X.R. (2009). Empirical assessment of the reproductive fitness components of the hybrid pine *Pinus densata* on the Tibetan Plateau. Evol. Ecol. **23**:447–462. https://doi.org/10.1007/s10682-008-9244-6.

Mao, K., Wang, Y., and Liu, J. (2021). Evolutionary origin of species diversity on the Qinghai–Tibet Plateau. J. Systemat. Evol. **59**:1142–1158. https://doi.org/10.1111/jse.12809.

Martin, B.T., Chafin, T.K., Douglas, M.R., and Douglas, M.E. (2021). ClineHelpR: an R package for genomic cline outlier detection and visualization. BMC Bioinf. **22**:501. https://doi.org/10.1186/s12859-021-04423-x.

Mayr, E. (1942). Systematics and the Origin of Species (Columbia University Press).

Menon, M., Bagley, J.C., Friedline, C.J., Whipple, A.V., Schoettle, A.W., Leal-Sàenz, A., Wehenkel, C., Molina-Freaner, F., Flores-Rentería, L., Gonzalez-Elizondo, M.S., et al. (2018). The role of hybridization during ecological divergence of southwestern white pine (*Pinus strobiformis*) and limber pine (*P. flexilis*). Mol. Ecol. **27**:1245–1260. https://doi.org/10.1111/mec.14505.

Milanesi, M., Capomaccio, S., Vajana, E., Bomba, L., Garcia, J.F., Ajmone-Marsan, P., and Colli, L. (2017). BITE: An R Package for Biodiversity Analyses. https://doi.org/10.1101/181610.

Mirov, N.T. (1967). The Genus *Pinus* (Ronald Press Company).

Moore, W.S. (1977). An evaluation of narrow hybrid zones in vertebrates. Q. Rev. Biol. **52**:263–277.

Neale, D.B., Wegrzyn, J.L., Stevens, K.A., Zimin, A.V., Puiu, D., Crepeau, M.W., Cardeno, C., Koriabine, M., Holtz-Morris, A.E., Liechty, J.D., et al. (2014). Decoding the massive genome of loblolly pine using haploid DNA and novel assembly strategies. Genome Biol. **15**:R59. https://doi.org/10.1186/gb-2014-15-3-r59.

Nei, M., and Li, W.H. (1979). Mathematical model for studying genetic variation in terms of restriction endonucleases. Proc. Natl. Acad. Sci. USA **76**:5269–5273. https://doi.org/10.1073/pnas.76.10.5269.

Neves, L.G., Davis, J.M., Barbazuk, W.B., and Kirst, M. (2013). Whole-exome targeted sequencing of the uncharacterized pine genome. Plant J. **75**:146–156. https://doi.org/10.1111/tpj.12193.

Nishizawa, A., Yabuta, Y., and Shigeoka, S. (2008). Galactinol and raffinose constitute a novel function to protect plants from oxidative damage. Plant Physiol. **147**:1251–1263. https://doi.org/10.1104/pp.108.122465.

Niu, S., Li, J., Bo, W., Yang, W., Zuccolo, A., Giacomello, S., Chen, X., Han, F., Yang, J., Song, Y., et al. (2022). The Chinese pine genome and methylome unveil key features of conifer evolution. Cell **185**:204–217.e14. https://doi.org/10.1016/j.cell.2021.12.006.

Nosil, P., Vines, T.H., and Funk, D.J. (2005). Prespective: reproductive isolation caused by natural selection against immigrants from divergent habitats. Evolution **59**:705–719. https://doi.org/10.1111/j.0014-3820.2005.tb01747.x.

Nosil, P., Funk, D.J., and Ortiz-Barrientos, D. (2009). Divergent selection and heterogeneous genomic divergence. Mol. Ecol. **18**:375–402. https://doi.org/10.1111/j.1365-294X.2008.03946.x.

Oksanen, J., Blanchet, F.G., Friendly, M., Kindt, R., Legendre, P., McGlinn, D., Minchin, P.R., O'Hara, R.B., Simpson, G.L., and Solymos, P. (2019). Vegan: community ecology package. R Package Version 2.5-6. 2019.

Opgenoorth, L., Vendramin, G.G., Mao, K., Miehe, G., Miehe, S., Liepelt, S., Liu, J., and Ziegenhagen, B. (2010). Tree endurance on the Tibetan Plateau marks the world's highest known tree line of the Last Glacial Maximum. New Phytol. **185**:332–342. https://doi.org/10.1111/j.1469-8137.2009.03007.x.

Oswald, J.A., Harvey, M.G., Remsen, R.C., Foxworth, D.U., Dittmann, D.L., Cardiff, S.W., and Brumfield, R.T. (2019). Evolutionary dynamics of hybridization and introgression following the recent colonization of Glossy Ibis (Aves: *Plegadis falcinellus*) into the New World. Mol. Ecol. **28**:1675–1691. https://doi.org/10.1111/mec.15008.

Petit, R.J., and Excoffier, L. (2009). Gene flow and species delimitation. Trends Ecol. Evol. **24**:386–393. https://doi.org/10.1016/j.tree.2009.02.011.

Petkova, D., Novembre, J., and Stephens, M. (2016). Visualizing spatial population structure with estimated effective migration surfaces. Nat. Genet. **48**:94–100. https://doi.org/10.1038/ng.3464.

Pickrell, J.K., and Pritchard, J.K. (2012). Inference of population splits and mixtures from genome-wide allele frequency data. PLoS Genet. **8**, e1002967. https://doi.org/10.1371/journal.pgen.1002967.

Price, A.L., Patterson, N.J., Plenge, R.M., Weinblatt, M.E., Shadick, N.A., and Reich, D. (2006). Principal components analysis corrects for stratification in genome-wide association studies. Nat. Genet. **38**:904–909. https://doi.org/10.1038/ng1847.

Purcell, S., Neale, B., Todd-Brown, K., Thomas, L., Ferreira, M.A.R., Bender, D., Maller, J., Sklar, P., de Bakker, P.I.W., Daly, M.J., and Sham, P.C. (2007). PLINK: a tool set for whole-genome association and population-based linkage analyses. Am. J. Hum. Genet. **81**:559–575. https://doi.org/10.1086/519795.

Raj, A., Stephens, M., and Pritchard, J.K. (2014). fastSTRUCTURE: variational inference of population structure in large SNP data sets. Genetics **197**:573–589. https://doi.org/10.1534/genetics.114.164350.

Ravinet, M., Faria, R., Butlin, R.K., Galindo, J., Bierne, N., Rafajlović, M., Noor, M.A.F., Mehlig, B., and Westram, A.M. (2017). Interpreting the genomic landscape of speciation: a road map for finding barriers to gene flow. J. Evol. Biol. **30**:1450–1477. https://doi.org/10.1111/jeb.13047.

Ren, G., Mateo, R.G., Liu, J., Suchan, T., Alvarez, N., Guisan, A., Conti, E., and Salamin, N. (2017). Genetic consequences of Quaternary climatic oscillations in the Himalayas: *Primula tibetica* as a case study based on restriction site-associated DNA sequencing. New Phytol. **213**:1500–1512. https://doi.org/10.1111/nph.14221.

Ryan, S.F., Fontaine, M.C., Scriber, J.M., Pfrender, M.E., O'Neil, S.T., and Hellmann, J.J. (2017). Patterns of divergence across the geographic and genomic landscape of a butterfly hybrid zone associated with a climatic gradient. Mol. Ecol. **26**:4725–4742. https://doi.org/10.1111/mec.14236.

Shannon, P., Markiel, A., Ozier, O., Baliga, N.S., Wang, J.T., Ramage, D., Amin, N., Schwikowski, B., and Ideker, T. (2003). Cytoscape: a software environment for integrated models of biomolecular interaction networks. Genome Res. **13**:2498–2504. https://doi.org/10.1101/gr.1239303.

Song, B.H., Wang, X.Q., Wang, X.R., Sun, L.J., Hong, D.Y., and Peng, P.H. (2002). Maternal lineages of *Pinus densata*, a diploid hybrid. Mol. Ecol. **11**:1057–1063. https://doi.org/10.1046/j.1365-294x.2002.01502.x.

Tajima, F. (1989). Statistical method for testing the neutral mutation hypothesis by DNA polymorphism. Genetics **123**:585–595. https://doi.org/10.1093/genetics/123.3.585.

Tian, L., Musetti, V., Kim, J., Magallanes-Lundback, M., and DellaPenna, D. (2004). The Arabidopsis *LUT1* locus encodes a member of the cytochrome p450 family that is required for carotenoid ε-ring hydroxylation activity. Proc. Natl. Acad. Sci. USA **101**:402–407. https://doi.org/10.1073/pnas.2237237100.

Todesco, M., Owens, G.L., Bercovich, N., Légaré, J.S., Soudi, S., Burge, D.O., Huang, K., Ostevik, K.L., Drummond, E.B.M., Imerovski, I., et al. (2020). Massive haplotypes underlie ecotypic differentiation in sunflowers. Nature **584**:602–607. https://doi.org/10.1038/s41586-020-2467-6.

Unte, U.S., Sorensen, A.M., Pesaresi, P., Gandikota, M., Leister, D., Saedler, H., and Huijser, P. (2003). *SPL8*, an SBP-box gene that affects pollen sac development in *Arabidopsis*. Plant Cell **15**:1009–1019. https://doi.org/10.1105/tpc.010678.

Van der Auwera, G.A., Carneiro, M.O., Hartl, C., Poplin, R., Del Angel, G., Levy-Moonshine, A., Jordan, T., Shakir, K., Roazen, D., Thibault, J., et al. (2013). From FastQ data to high confidence variant calls: the Genome Analysis Toolkit best practices pipeline. Curr. Protoc. Bioinformatics **43**:11.10.1–11.10.33. https://doi.org/10.1002/0471250953.bi1110s43.

Wang, B., Mao, J.F., Gao, J., Zhao, W., and Wang, X.R. (2011). Colonization of the Tibetan Plateau by the homoploid hybrid pine *Pinus densata*. Mol. Ecol. **20**:3796–3811. https://doi.org/10.1111/j.1365-294X.2011.05157.x.

Wang, G., Oh, D.H., and Dassanayake, M. (2020). GOMCL: a toolkit to cluster, evaluate, and extract non-redundant associations of Gene Ontology-based functions. BMC Bioinf. **21**:139. https://doi.org/10.1186/s12859-020-3447-4.

Wang, X.R., and Szmidt, A.E. (1994). Hybridization and chloroplast DNA variation in a *Pinus* species complex from Asia. Evolution **48**:1020–1031. https://doi.org/10.1111/j.1558-5646.1994.tb05290.x.

Wen, J., Zhang, J.Q., Nie, Z.L., Zhong, Y., and Sun, H. (2014). Evolutionary diversifications of plants on the Qinghai-Tibetan Plateau. Front. Genet. **5**:4. https://doi.org/10.3389/fgene.2014.00004.

Willyard, A., Syring, J., Gernandt, D.S., Liston, A., and Cronn, R. (2007). Fossil calibration of molecular divergence infers a moderate mutation rate and recent radiations for *Pinus*. Mol. Biol. Evol. **24**:90–101. https://doi.org/10.1093/molbev/msl131.

Wu, C.L. (1956). The taxonomic revision and phytogeographical study of Chinese pines. Acta Phytotaxon. Sin. **5**:131–163.

Wu, C.I. (2001). The genic view of the process of speciation. J. Evol. Biol. **14**:851–865. https://doi.org/10.1046/j.1420-9101.2001.00335.x.

Xia, H., Wang, B., Zhao, W., Pan, J., Mao, J.F., and Wang, X.R. (2018). Combining mitochondrial and nuclear genome analyses to dissect the effects of colonization, environment, and geography on population structure in *Pinus tabuliformis*. Evol. Appl. **11**:1931–1945. https://doi.org/10.1111/eva.12697.

Zhao, W., Sun, Y.Q., Pan, J., Sullivan, A.R., Arnold, M.L., Mao, J.F., and Wang, X.R. (2020). Effects of landscapes and range expansion on population structure and local adaptation. New Phytol. **228**:330–343. https://doi.org/10.1111/nph.16619.

Zhao, W., Meng, J., Wang, B., Zhang, L., Xu, Y., Zeng, Q.Y., Li, Y., Mao, J.F., and Wang, X.R. (2014). Weak crossability barrier but strong juvenile selection supports ecological speciation of the hybrid pine *Pinus densata* on the Tibetan Plateau. Evolution **68**:3120–3133. https://doi.org/10.1111/evo.12496.

Zheng, B., Xu, Q., and Shen, Y. (2002). The relationship between climate change and Quaternary glacial cycles on the Qinghai–Tibetan Plateau: review and speculation. Quat. Int. **97-98**:93–101. https://doi.org/10.1016/s1040-6182(02)00054-x.

Plant Communications, Volume *4*

Supplemental information

Genomic clines across the species boundary between a hybrid pine and its progenitor in the eastern Tibetan Plateau

Jing-Fang Guo, Wei Zhao, Bea Andersson, Jian-Feng Mao, and Xiao-Ru Wang

Supplemental Information

Genomic clines across the species boundary between a hybrid pine and its progenitor in the eastern Tibetan Plateau

Jing-Fang Guo, Wei Zhao, Bea Andersson, Jian-Feng Mao, Xiao-Ru Wang

Supplemental information for fastsimcoal analysis

METHOD

As a complementary test to the Stairway Plot approach, we inferred the demographic history of *P. densata* groups (E, C, W, SW) using a coalescent simulation-based method fastsimcoal2 version 2.7 (Excofffier *et al.*, 2021) which takes divergence and migration among populations into consideration. Complex multi-group fastsimcoal models that include migration and population size changes are computationally very time consuming. Previous study (Gao *et al.*, 2012) and our PCA and Stairway results show that group SW and W diverged rather recently, are genetically close and share a similar demography history. Thus to simplify the models, we combined W and SW as one group for this analysis. Because missing data can bias the estimates of SFS, we used a down-sampling procedure to generate the folded SFS using a Python script available on Dryad (Papadopoulou & Knowles, 2015) with minor modification (https://github.com/hui-liu/Bioinformatics-Scripts/blob/master/Scripts/Python/sampleDownMSFS_Hui_final.py). The final folded SFS included low-frequency alleles and invariants.

We tested 11 scenarios, all of which involved the ancestral population splitting into three different groups with gene flow (gene flow was not shown in the Figure 1) but differed in terms of: (i) levels of gene flow between the groups; (ii) the mode of population size change after the split, (iii) whether strong bottleneck occurred or not, and (iv) when the bottleneck occurred (Fig. 1).

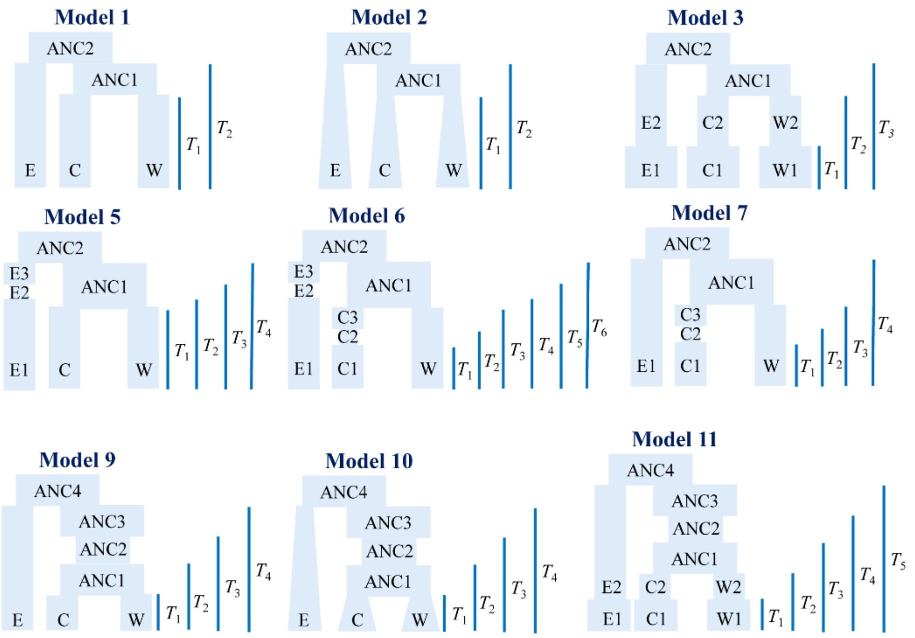

Fig. 1 The 11 tested demographic models.

Each model was run 50 independent times, with 100,000 coalescent simulations as well as 40 expectation-conditional maximization cycles for the calculation of the global maximum-likelihood. The best-fitting model was selected based on the maximum value of likelihood over the 50 independent runs of each modle and their Akaike's weight of evidence (Excoffier *et al.*,

2013). The goodness of fit of the best model was tested by comparing the observed SFS with the expected SFS, which was obtained by 100,000 coalescent simulations under the maximum-likelihood estimates of population parameters. We assumed a mutation rate of 7×10^{-10} mutation per site per year and a generation time of 50 years for the genus *Pinus* (Willyard *et al.*, 2007) to convert model parameters to absolute values.

RESULTS

Of the 11 demographic models, the best-fitting model was model 10. The expected SFS from this model projected the observed SFS well (Fig. 2), suggesting that the demographic parameters recovered are good estimates of the past population history. The model estimated a divergence time of 5.36 million years ago between the E and the ancestor population of group C and W+SW (Fig. 3). The estimated effective population size (N_e) of the common ancestor of the three groups was 1.4×10^4, and N_e of the common ancestor of group C and W+SW was 2.9×10^4. At 0.88–0.58 MYA, this ancestral population of group C and W+SW experienced a strong bottleneck, during which the N_e reduced to half of the original size, then followed by an instantaneous expansion at ~ 0.58 MYA. The group C and W+SW diverged ~ 0.12 MYA accompanied by a bottleneck in each resulting group. The W+SW stared with a very small population size ~1000 individuals. All groups expanded recently.

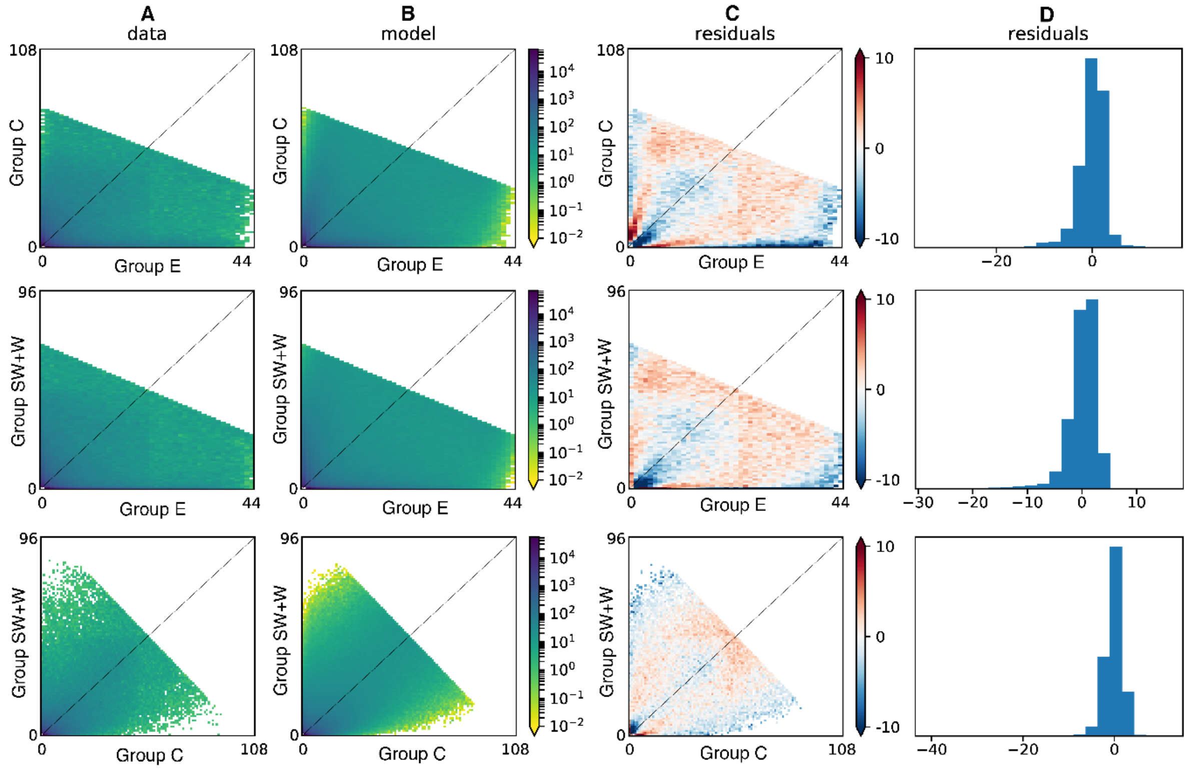

Fig. 2 The goodness of fit of the best-fitting demographic model inferred by fastsimcoal2. For each pair of groups, group E *vs*. C (top), group E *vs*. SW+W (middle) and group C *vs*. SW+W (bottom), the observed joint SFS (data, A) is compared with expected joint SFS (model, B), and the residuals between data and model are plotted in a colormap (C) and a histogram (D). In the colormap, red or blue residuals indicate that the model predicts too many to too few alleles in a given cell, respectively.

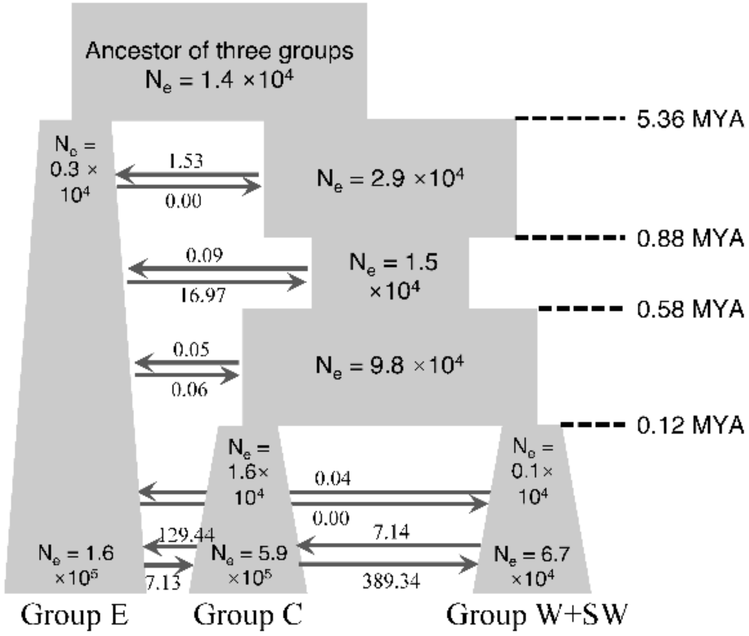

Fig. 3 Demographic model of divergence in *Pinus densata*. Each block represents a current or ancestral population with their estimated effective population size (N_e). Arrows indicate the direction of gene flow with the estimated migration rate labeled above or below the arrow. The timing of population splits and population size changes are indicated in million years ago (MYA).

REFERENCES

Excofffier L, Marchi N, Marques DA, Matthey-Doret R, Gouy A, Sousa VC. 2021. fastsimcoal2: demographic inference under complex evolutionary scenarios. *Bioinformatics* **37**: 4882-4885.

Excoffier L, Dupanloup I, Huerta-Sánchez E, Sousa VC, Foll M. 2013. Robust demographic inference from genomic and SNP data. *PLoS Genetics* **9**: e1003905.

Gao J, Wang B, Mao JF, Ingvarsson P, Zeng QY, Wang XR. 2012. Demography and speciation history of the homoploid hybrid pine *Pinus densata* on the Tibetan Plateau. *Molecular Ecology* **21**: 4811-4827.

Papadopoulou A, Knowles LL. 2015. Genomic tests of the species-pump hypothesis: recent island connectivity cycles drive population divergence but not speciation in Caribbean crickets across the Virgin Islands. *Evolution* **69**: 1501-1517.

Willyard A, Syring J, Gernandt DS, Liston A, Cronn R. 2007. Fossil calibration of molecular divergence infers a moderate mutation rate and recent radiations for *Pinus*. *Molecular Biology and Evolution* **24**: 90-101.

Supplemental Figures

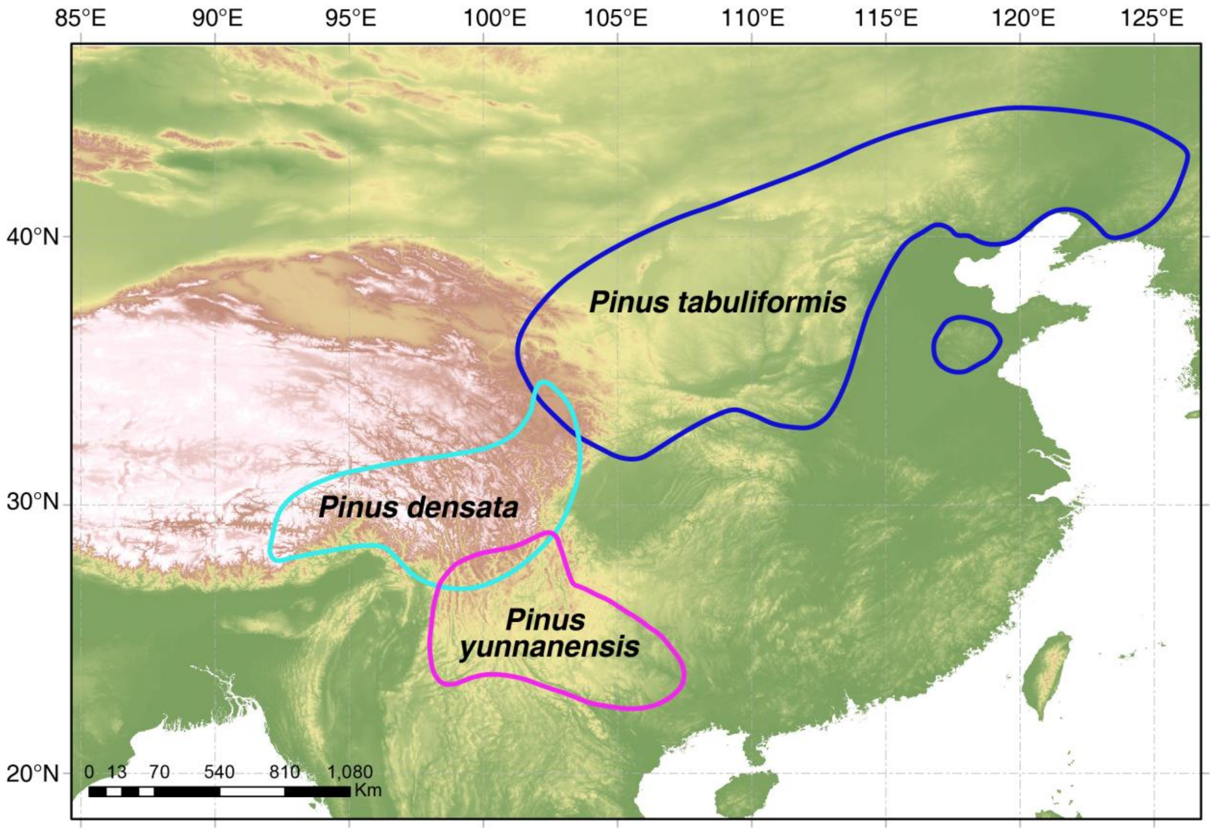

Fig. S1 Distribution map of *Pinus densata*, *P. tabuliformis* and *P. yunnanensis* (adapted from Mao and Wang, 2011, American Naturalist 177:424-439).

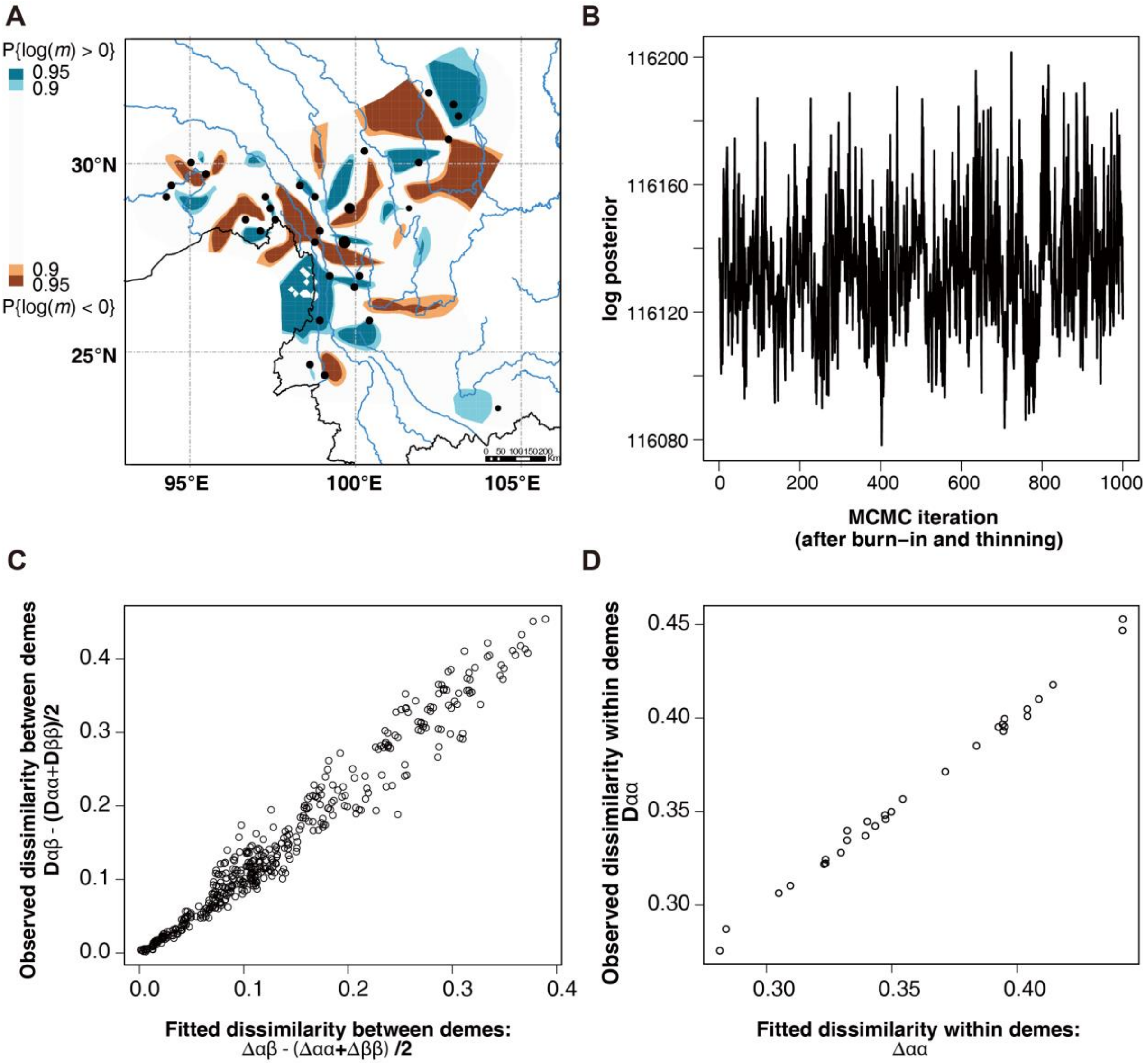

Fig. S2 The reliability of the estimated effective migration surface (EEMS) models. (A) The posterior probabilities of estimated effective migration surface in *Pinus densata* and *P. yunnanensis*. Brown contours represent areas of low relative migration supported by posterior probabilities >0.9. Blue contours represent areas of high relative migration supported by posterior probabilities >0.9. Size of dots reflects numbers of samples in a merged locality. (**B**) Posterior probability trace showing the convergence of EEMS. Observed versus fitted dissimilarities within-demes component (**C**) and between-demes component (**D**). Strong linear relationship between the observed and fitted values indication good fit of EEMS model.

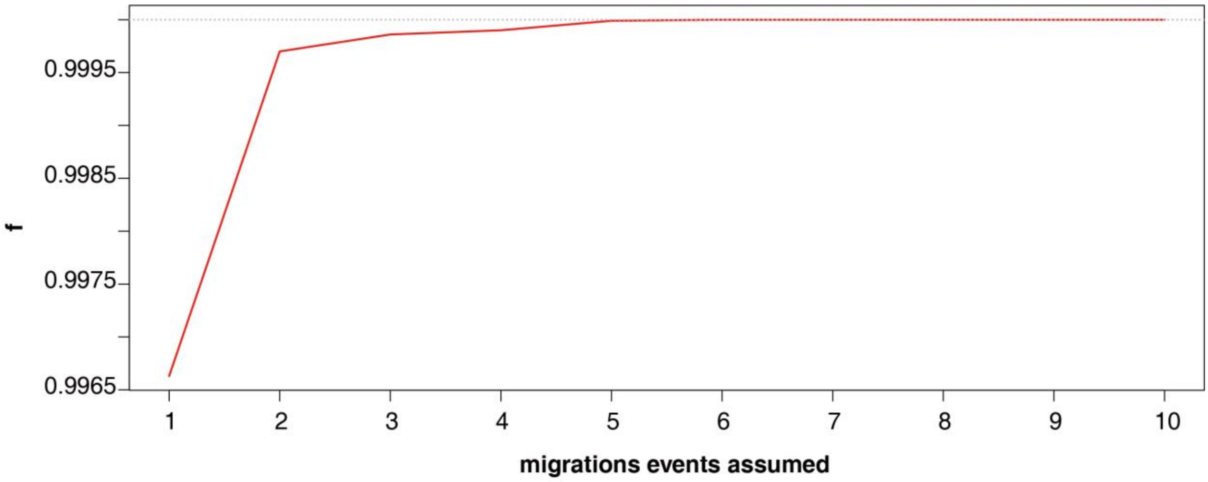

Fig. S3 Measuring models fit in Treemix. Plot showing the *f*-index, which represents the fraction of the variance in the sample covariance matrix explained by the model covariance matrix, as criteria for model fitting for the number of migration events ranging from 1 to 10.

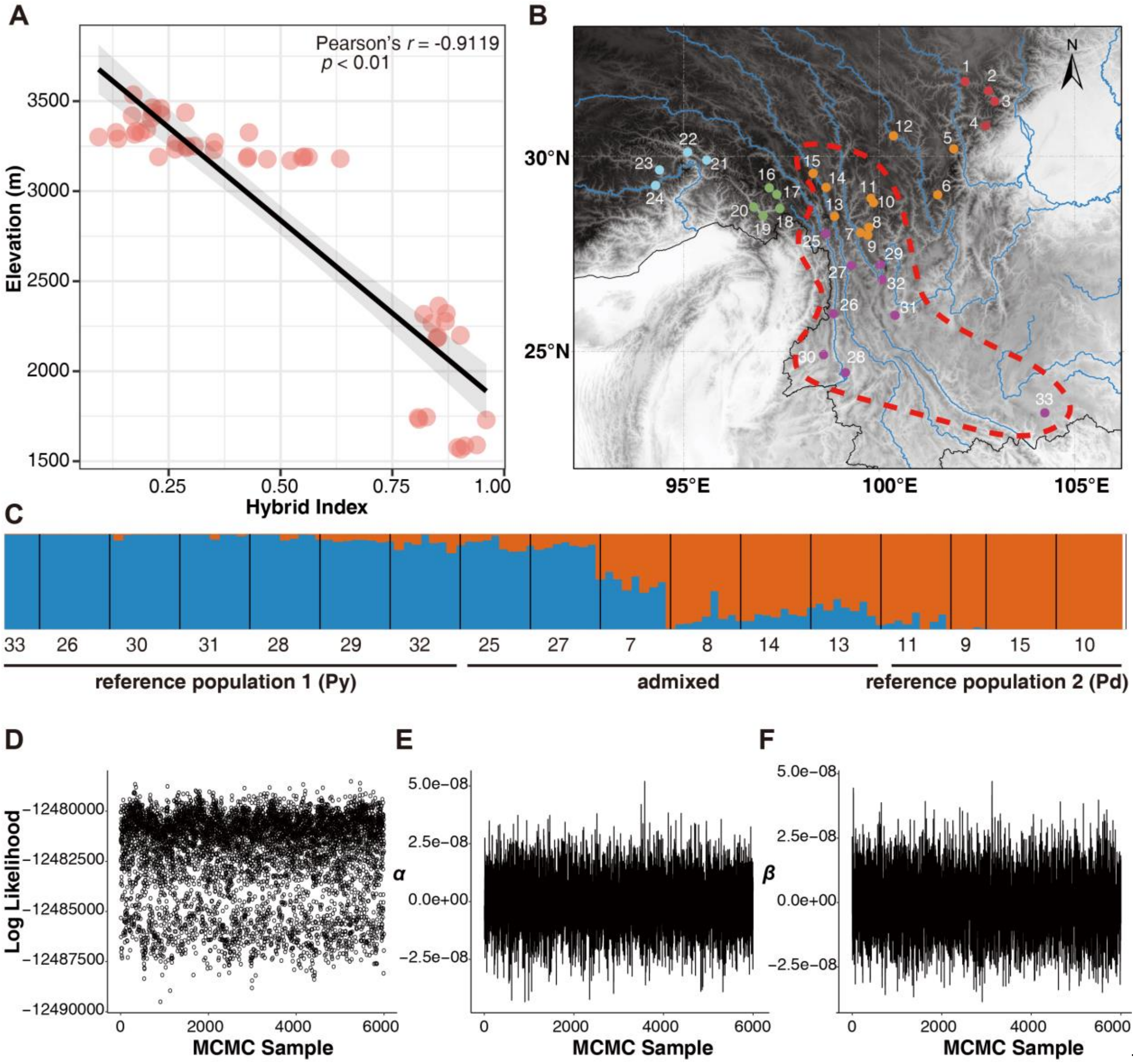

Fig. S4 A zone of species transition selected for Bayesian estimation of genomic clines (BGC) analysis, and the reliability of the BGC models. (A) Correlation of hybrid index of admixed individuals with elevation. **(B)** The distribution of the 17 populations for BGC analysis are circled with red dashes. **(C)** ADMIXTURE analysis of the 17 populations. Inspection of the traces by aggregating three independent runs of BGC. Scatter-plot shows the log likelihood **(D)**, α **(E)**, and β **(F)** of MCMC samples.

Supplemental Tables

Table S1 Differiatation (F_{ST}) between and within the group (E, C, SW and W) of *Pinus densata*.

	Pd-E	Pd-C	Pd-SW	Pd-W
Pd-E	0.0453			
Pd-C	0.2031	0.0269		
Pd-SW	0.2622	0.0832	0.0294	
Pd-W	0.2935	0.1171	0.0966	0.0414

Note: Within-group F_{ST} is in red on the diagonal.

Table S2 Introgression events supported by *D* and f_4 statistics calculated by Dsuite.

P1	P2	P3	*D* statistic	Z-score	*P*-value	*f*4-ratio	BBAA	ABBA	BABA
Pt	E	C	0.0899282	27.3914	0	0.132896	4426.8	4094.13	3418.53
C	SW	Py	0.0494395	10.2406	0	0.270799	3580.85	3672.5	3326.48
Pt	E	Py	0.0845899	24.003	0	0.104286	4509.01	4066.91	3432.54
Pt	E	SW	0.0911228	25.4913	0	0.102746	4467.87	4093.17	3409.5
Pt	E	W	0.0925236	25.231	0	0.093165	4452.32	4086.35	3394.22
W	SW	Py	0.0864658	17.1188	0	0.362799	4352.21	3334.25	2803.54
W	C	Py	0.0271345	4.94543	7.60E-07	0.126251	3774.36	3495.45	3310.76
Py	C	E	0.0200026	4.28036	1.87E-05	0.16918	6850.76	2789.94	2680.51
Py	C	Pt	0.0131173	2.72075	0.00651338	0.0596882	7369.89	2633.77	2565.57
Py	SW	E	0.012839	2.31878	0.0204071	0.104096	7108.39	2659.54	2592.11
Py	W	E	0.0137577	2.10117	0.0356259	0.117647	6715.09	2805.68	2729.53
SW	C	Pt	0.0099593	1.85922	0.0629964	0.043718	7530.55	2540.11	2490.02
W	C	Pt	0.00999736	1.77899	0.0752407	0.0442488	7532.34	2517.35	2467.52
SW	C	E	0.00793074	1.6468	0.0995991	0.07213	6983.96	2668.77	2626.77
W	C	E	0.00634253	1.20105	0.229733	0.0581827	6979.3	2639.56	2606.29
SW	W	C	0.0039803	0.931159	0.351772	0.267623	4180.83	3095.75	3071.2
Py	SW	Pt	0.00362587	0.609817	0.541983	0.0155942	7637.82	2505.63	2487.52
Py	W	Pt	0.00349053	0.52439	0.600007	0.0160729	7241.18	2639.95	2621.59
SW	W	E	0.00186384	0.375315	0.707426	0.0149991	7803.24	2345.15	2336.43
SW	W	Pt	5.86E-05	0.0110131	0.991213	0.000229053	8377.14	2227.3	2227.04

Notes: Only comparisons that are compatible with the inferred phylogenetic relationships and result in positive *D* values are shown.

BBAA: number of sites at which species P1 and P2 shared the derived allele.

ABBA: number of sites at which species P2 and P3 shared the derived allele.

BABA: number of sites at which species P1 and P3 shared the derived allele.

Table S6 Environmental parameters used in this study.

Code	Name	Source	Resultion
bio1	Annual mean air temperature	https://www.climond.org/	30 arc-seconds
bio3	Isothermality	https://www.climond.org/	30 arc-seconds
bio4	Air temperature seasonality	https://www.climond.org/	30 arc-seconds
bio12	Annual precipitation	https://www.climond.org/	30 arc-seconds
bio14	Precipitation of the driest month	https://www.climond.org/	30 arc-seconds
bio15	Precipitation seasonality	https://www.climond.org/	30 arc-seconds
FRS	Ground-frost frequency	http://www.ipcc-data.org/observ/clim/cru_climatologies.html	30 arc-seconds
GDD	Growing degree days	http://nelson.wisc.edu/sage/data-and-models/atlas/maps.php	30 arc-seconds
SC	Soil organic carbon	http://nelson.wisc.edu/sage/data-and-models/atlas/maps.php	30 arc-seconds
WET	Wet-day frequency	http://www.ipcc-data.org/observ/clim/cru_climatologies.html	30 arc-minutes
UVB1	Annual mean UV-B	http://www.ufz.de/gluv/	15 arc-minutes
